# Carcinoembryonic antigen cell adhesion molecule 6 (*CEACAM6*) in Pancreatic Ductal Adenocarcinoma (PDA): *An integrative analysis of a novel therapeutic target*

**DOI:** 10.1038/s41598-019-54545-9

**Published:** 2019-12-04

**Authors:** Ritu Pandey, Muhan Zhou, Shariful Islam, Baowei Chen, Natalie K Barker, Paul Langlais, Anup Srivastava, Moulun Luo, Laurence S. Cooke, Eric Weterings, Daruka Mahadevan

**Affiliations:** 1 University of Arizona Cancer Center, University of Arizona, Tucson, USA; 2 Department of Cellular and Molecular Medicine, University of Arizona, Tucson, USA; 3 Department of Medicine, College of Medicine, University of Arizona, Tucson, USA; 4 Department of Radiation Oncology, College of Medicine, University of Arizona, Tucson, USA

**Keywords:** Pancreatic cancer, Biomarkers

## Abstract

We investigated biomarker CEACAM6, a highly abundant cell surface adhesion receptor that modulates the extracellular matrix (ECM) in pancreatic ductal adenocarcinoma (PDA). The Cancer Genome Atlas (TCGA) and Gene Expression Omnibus (GEO) RNA-Seq data from PDA patients were analyzed for CEACAM6 expression and evaluated for overall survival, association, enrichment and correlations. A CRISPR/Cas9 Knockout (KO) of CEACAM6 in PDA cell line for quantitative proteomics, mitochondrial bioenergetics and tumor growth in mice were conducted. We found CEACAM6 is over-expressed in primary and metastatic basal and classical PDA subtypes. Highest levels are in classical activated stroma subtype. CEACAM6 over-expression is universally a poor prognostic marker in *KRAS* mutant and wild type PDA. High CEACAM6 expression is associated with low cytolytic T-cell activity in both basal and classical PDA subtypes and correlates with low levels of T-_REG_ markers. In HPAF-II cells knockout of CEACAM6 alters ECM-cell adhesion, catabolism, immune environment, transmembrane transport and autophagy. CEACAM6 loss increases mitochondrial basal and maximal respiratory capacity. HPAF-II CEACAM6−/− cells are growth suppressed by >65% vs. wild type in mice bearing tumors. CEACAM6, a key regulator affects several hallmarks of PDA including the fibrotic reaction, immune regulation, energy metabolism and is a novel therapeutic target in PDA.

## Introduction

Carcinoembryonic antigen cell adhesion molecule 6 (CEACAM6) is a cell adhesion receptor of the immunoglobulin-like superfamily (3 Ig-like domains), known to interact with other CEACAMs^1–4^ through *cis* and *trans* forming dimers via their N-terminal IgG V-domain^1^. CEACAM6 is anchored to the cell membrane via a glycophosphatidylinositol (GPI) anchor at its C terminus and regulates cell adhesion, proliferation, signaling in cancer, and immunity. CEACAM6 elaborates an extracellular matrix (ECM) interactome via homotypic and/or heterotypic binding, promoting fibronectin (FN1)-integrin (ITGA1 and ITGB1) interactions^5^. Over-expression of CEACAM6 is documented in many human epithelial (e.g. colorectal, breast, pancreatic ductal adenocarcinoma (PDA))^6,7^ and hematologic malignancies (e.g. multiple myeloma and acute lymphoblastic leukemia)^5^.

In human epithelial carcinomas, CEACAM6 over-expression leads to *anoikis*, a regulated cell death mechanism induced by inadequate or inappropriate cell-matrix interactions via an ECM interaction, promoting invasion^8^. PDA progression is accompanied by a fibrotic stromal desmoplastic reaction (DR) due to an extensive deposition of ECM components intermingled with pancreatic stellate cells, a reduced vasculature, a suppressed immune-surveillance and a hypoxic altered metabolic status^4,9^. In PDA, CEACAM6 over-expression plays a role in reshaping the ECM-cell adhesion processes that promote anoikis resistance^10^.

Recent analyses of PDA datasets using bioinformatics methods have made it possible to classify tumor and stromal cell types using molecular stratification based on distinct features^11–13^. These studies have provided multiple subtypes with different classifications but with overlapping molecular-cellular characteristics for PDA. Immunologically, PDA is characterized by a highly suppressive immune tumor microenvironment (TME) with a sparse T-cell infiltration^14^. It has been established that PDA with normal stroma types (a small subset of patients) have comparatively high cytolytic T-cell activity, and are enriched for immune gene programs, whereas classical subtypes with activated stroma have very low T-cell cytolytic activity^15^.

We performed an integrative analysis of CEACAM6, a predictive biomarker that has not been investigated thoroughly using genome, proteome and functional studies as a potential candidate therapeutic target in PDA. We conducted a detailed analysis by expression profiling of CEACAM6 in several PDA types, tumor-stromal cells, and PDA cell lines to elucidate expression across PDA tumor types, effect on survival, association with the stroma, immune environment, relevance to *KRAS* mutations, proteomics and tumor growth potential of CECACAM6 knockout in PDA cells.

## Results

### CEACAM6 is over-expressed in PDA but is differentially expressed across subtypes

**Figure 1:**
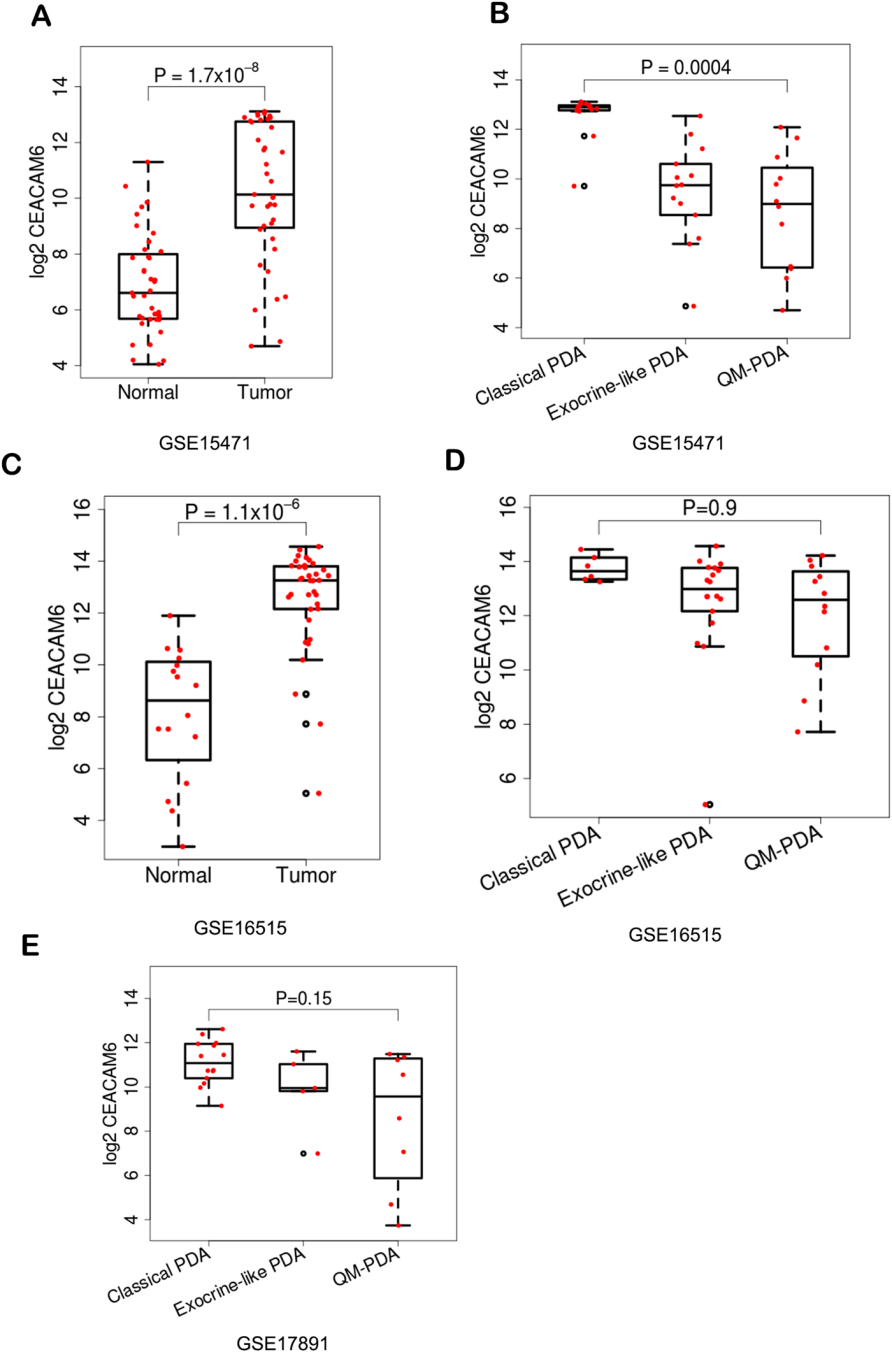
Box plots showing expression of CEACAM6 between normal and PDA patient samples from (**A**) GSE15471 and (**C**) GSE16515. Differential expression of CEACAM6 across subtypes – classical, QM and exocrine in (**B**) GSE15471 (**D**) GSE16515 and (**E**) GSE17891.

We analyzed expression datasets from GEO, TCGA and ICGC to evaluate the expression of CEACAM6. Independent analysis of expression array and RNA-seq datasets from GEO and TCGA datasets was carried out for PDA tumors. Tumor and normal samples were compared as a group and as paired samples when available from GSE15471, GSE16515 and GSE17891. All tumor samples were obtained at the time of surgery from resected PDA patients. Our assessment of expression trends across datasets showed that in any PDA cohort, approximately ~80% of patient samples have an elevated expression of CEACAM6. Compared to normal cells, CEACAM6 is 10 to 20-fold higher in PDAs (Fig. 1A,C). Recent studies^11–13^, have classified PDA into subtypes based on gene expression profiling and CEACAM6 is one of the most significant genes changing in these studies. These types fall broadly into three categories, a) Classical or Pancreatic Progenitor, b) Quasi-mesenchymal (QM) or basal like and c) Exocrine like. We evaluated four studies with different subtypes. Within the subtypes of PDA, as defined by^11^, we identified CEACAM6 expression to be much higher in classical than the QM subtype and a relative intermediate level in exocrine samples^11,16,17^, (Fig. 1B,D,E). In the ICGC dataset we found CEACAM6 to be high in >90% of samples in all subtypes (Supplementary Fig. 1) with highest expression in the classical subtype as noted before.

**Figure 2:**
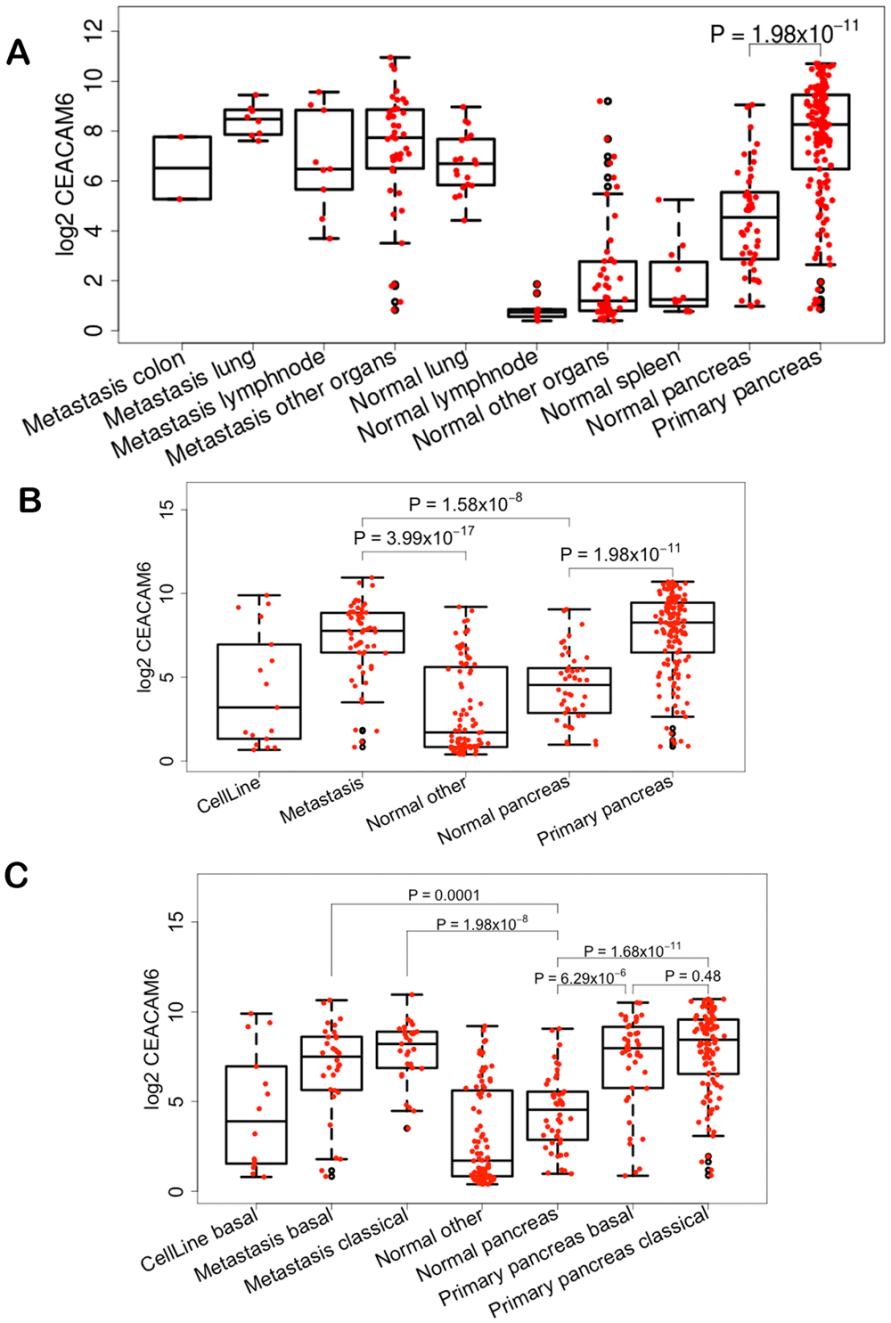
Box plots of expression of CEACAM6 from GSE71729 showing relative expression (**A**) across normal, primary and metastatic tumors from different organs, (**B**) across PDA cell lines, normal and primary PDA, (**C**) across basal and classical subtypes.

**Figure 3:**
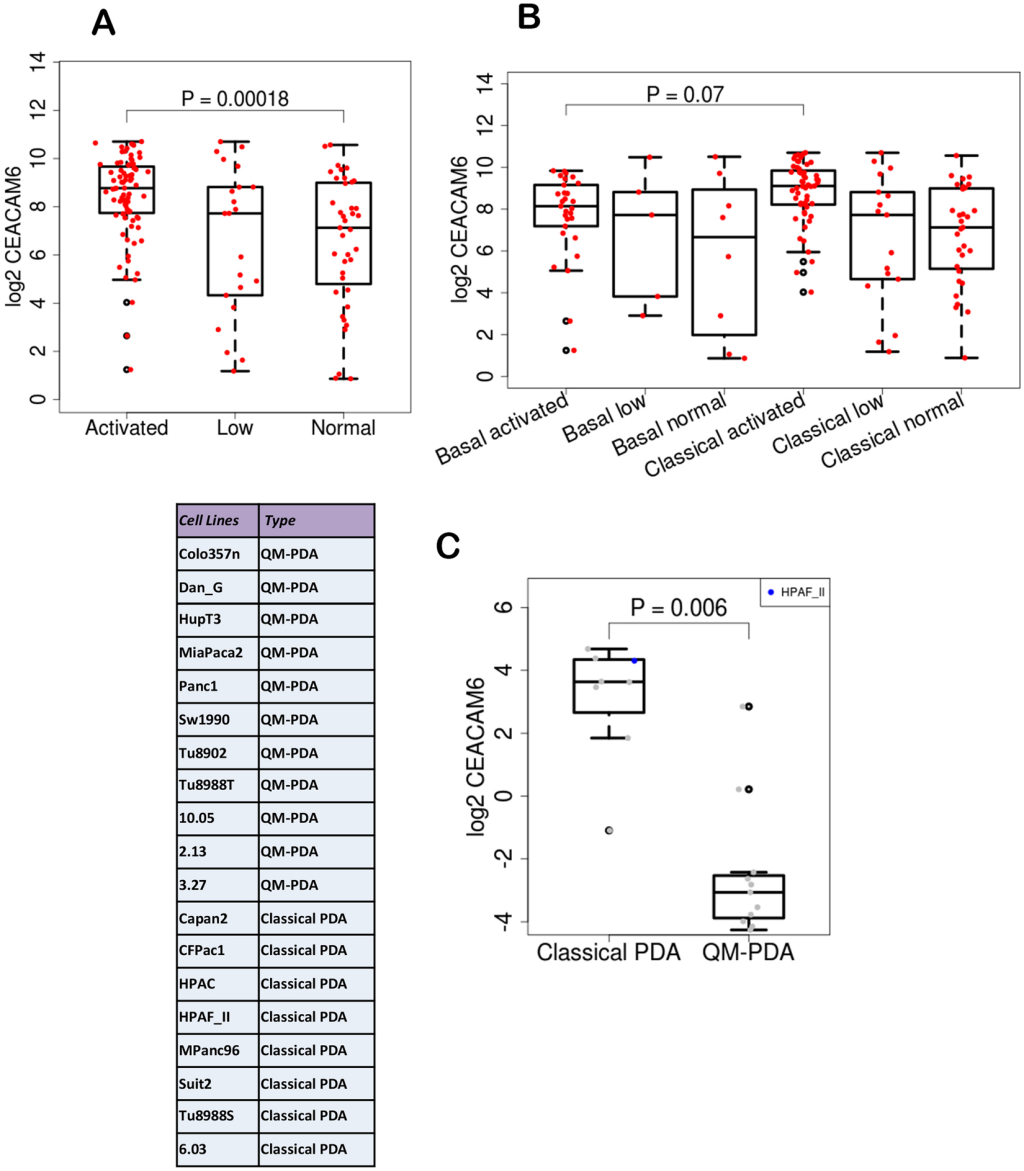
CEACAM6 expression (**A**) across normal and activated stroma types, (**B**) across activated and normal stroma within classical and basal subtypes in GSE71729. (**C**) Classification of PDA cell lines by QM or classical and CEACAM6 expression across the cell lines that belong to two types of PDA.

Analysis of subtypes in the^12^ dataset showed that CEACAM6 expression is elevated across primary and metastatic subtypes of many PDAs compared to normal samples from multiple organ types (Fig. 2A). In PDA, it is elevated in primary and metastatic disease compared to normal pancreas (Fig. 2B). Further, within PDA it is elevated both in primary basal, primary classical, metastatic basal and metastatic classical, but is highest in the classical subtypes (Fig. 2C). We also analyzed the CEACAM6 levels across stroma types defined by^12^ and found that it is significantly elevated in activated stroma compared to low and normal stroma (Fig. 3A). Stratifying the samples by basal and classical subtypes showed CEACAM6 has the highest level of expression in activated stroma in the classical subtype (Fig. 3B). It is well established that ~50% of human PDA cell lines do not express CEACAM6. In addition, several PDA cell lines are classified as QM and classical types^11^. Fig. 3C, shows the expression of CEACAM6 in the two types of PDA. We show that QM type cell lines do not express CEACAM6, whereas it is over-expressed in the classical types. This correlates well with the pattern of expression observed in PDA patients where the QM subtypes have a relatively lower level of CEACAM6 expression compared to the classical subtype. Overall CEACAM6 has a significantly high expression across human PDA and is a unique molecule as it is absent in mice (e.g. KPC, KRAS-LSL) that are widely used for PDA studies. CEACAM6 gene is present in higher mammals such as monkeys with the highest sequence identity to humans being found in the macaques.

### High CEACAM6 expression has a negative influence on overall survival in PDA patients

**Figure 4:**
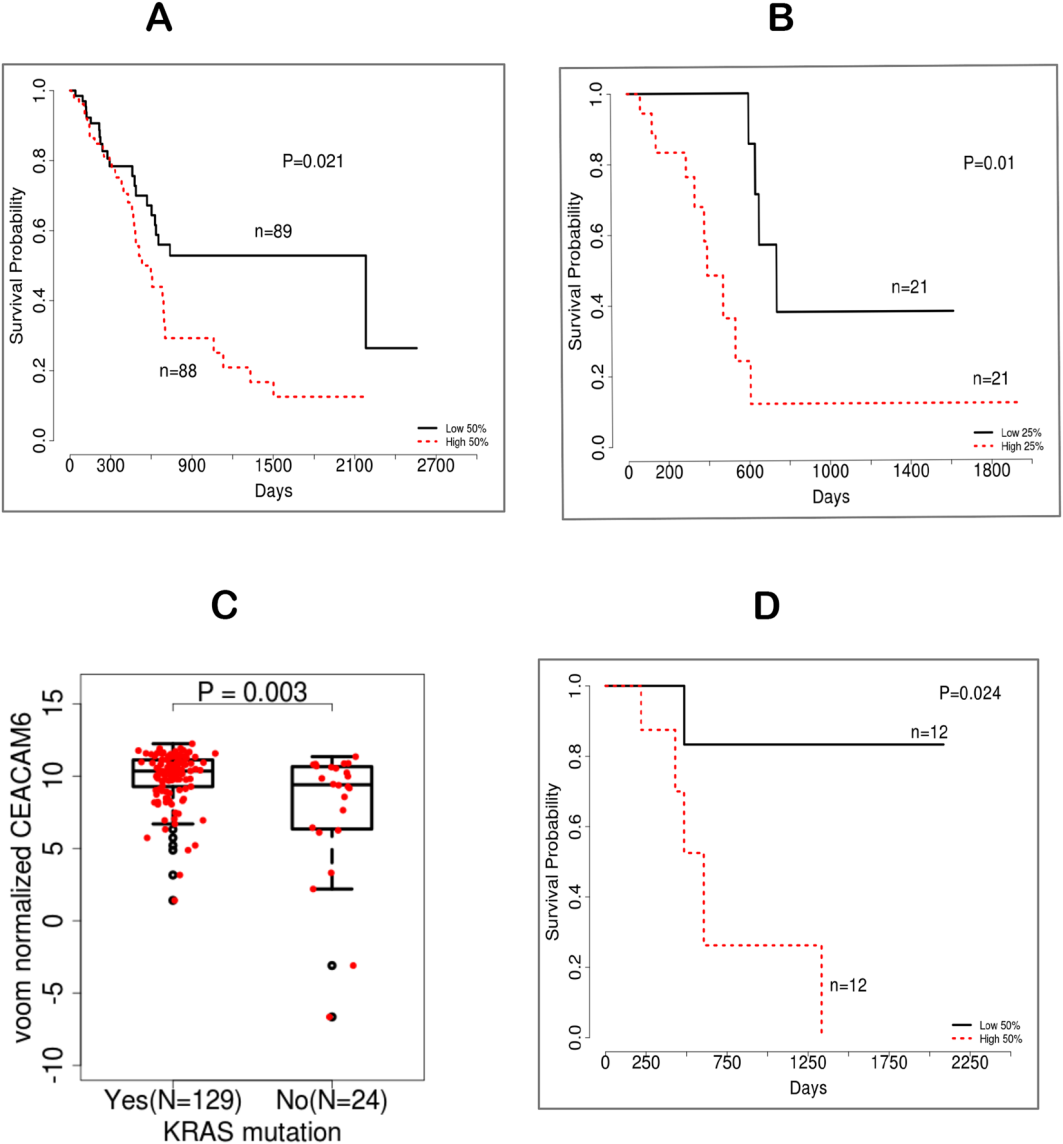
(**A**) Kaplan-Meier survival analysis of TCGA PDA patients stratified by median CEACAM6 expression showing differential prognosis between the two cohort (P = 0.02), log-rank test. (**B**) Survival analysis of classical type samples stratified by CEACAM6 median expression. (**C**) Box plot showing differential expression of TCGA PDA samples with and without mutant *KRAS*. (**D**) Survival analysis of PDA patients in absence of mutant *KRAS* stratified by CEACAM6 median expression.

We analyzed the TCGA dataset for overall survival (OS) of PDA patients stratified by CEACAM6 expression. A median survival analysis shows that PDA patients with high expression of CEACAM6 have poor prognosis than those with lower expression (Fig. 4A). The survival distributions between high (>50%) and low (<50%) CEACAM6 PDA samples are significantly different (log-rank test p = 0.021). The cox regression model indicates that the expected hazard of the high >50% CEACAM6 is 1.92 times higher with a 95% CI of (1.11, 3.30) as compared to the low <50% CEACAM6 group. It has been established that subtypes provide prognostic information with regard to OS in PDA resection samples and the classical subtype fairs better than the basal subtype^12^. CEACAM6 is expressed highly across both types but relatively higher in classical. In Fig. 4A, both high and low CEACAM6 groups contain classical and basal samples, but the high CEACAM6 has more classical samples than the basal. We assessed OS within the classical subtype with regard to high CEACAM6 expression for patient prognosis (Fig. 4B). Since CEACAM6 expression is high across many samples we took 25% top and bottom CEACAM6 expression samples for analysis. The survival distribution between high CEACAM6 (>25%) and low (<25%) CEACAM6 in classical TCGA samples are significantly different (log-rank test p = 0.018). Our survival analysis showed that within the classical subtype, samples with high CEACAM6 expression have a poor survival trend versus low CEACAM6 expression. Analysis from^11^ also shows the same trend although sample size is limited to provide better estimates (Supplementary Fig. 2A)^12^, have shown that normal stroma has better survival than activated stroma type in PDA. We evaluated the same data and performed survival analysis of stratified samples by median high and low CEACAM6 expression and found that, in activated stroma, there is no differential survival, which is expected since CEACAM6 is mostly highly expressed in activated stroma and overall the PDA samples with activated stroma have a poor survival. In normal stroma, dividing the population into high and low, shows poor OS for patients with high CEACAM6, almost the same as patients with activated stroma. In contrast, patients with low CEACAM6 expression have a better overall survival trend than the other subtypes (Supplementary Fig. 2B).

### CEACAM6 is a poor prognostic marker in KRAS mutant and wild type PDA

To investigate if CEACAM6 expression is associated with samples with mutant *KRAS*, we analyzed TCGA RNA-Seq dataset of PDA samples that constitute 129 mutant *KRAS* and 24 WT *KRAS* samples. Differential analysis between mutant and WT samples was performed. Analysis of these tumors show that samples with mutant *KRAS* have high CEACAM6 expression whereas WT *KRAS* have a relatively low CEACAM6 expression (Fig. 4C) with a statistically significant fold difference and p-value.

A Chi-square test was performed to assess the significance of CEACAM6 association with mutant *KRAS* samples (Supplementary Table 1). In the analysis, 89.4% samples in the 50% high CEACAM6 group had a *KRAS* mutation, while 78.3% samples in the 50% low CEACAM6 group had a *KRAS* mutation (p = 0.09). Comparing the top 25% CEACAM6 expression and the rest of the samples, the distribution of mutant *KRAS* has the same direction and the difference is statistically significant (p = 0.03). The 25% high CEACAM6 samples have more mutant *KRAS* than the lower CEACAM6 expresser samples. The classical PDA subtype has been shown to be dependent on mutant *KRAS*. A study reported earlier^18^ with multiple cancer cell lines for gene signatures in *KRAS* dependent cells showed CEACAM6 as one of the genes associated or dependent on *KRAS*. We also found a significant association with a p-value 0.002 between continuous high CEACAM6 expression and mutant *KRAS* PDA samples using logistic regression. We did not find any significant survival outcome differences based on CEACAM6 expression levels in *KRAS* mutant samples as expected (Supplementary Fig. 2C). We investigated CEACAM6 expression level and outcome in *KRAS* WT patients. Figure 4D demonstrates the survival curves by CEACAM6 high 50% and low 50% expression groups in *KRAS* WT samples. Patients with high CEACAM6 have a poorer overall outcome than the patients with low CEACAM6 expression that is almost comparable to the outcome of *KRAS* mutant patient samples (Supplementary Fig. 2C).

### CEACAM6 high expression is associated with Immune suppression

The classical PDA have low cytolytic T-cell activity compared to basal and squamous subtypes with comparatively high cytolytic T-cell activity, a higher T-cell infiltration with increased immune markers^15^. Classical PDA subtypes are at a disadvantage in that immune checkpoint therapies are less likely to be effective as has been observed in clinical trials^19^. We investigated TCGA for high and low cytolytic T-cell activity with our stratified CEACAM6 high and low samples. Using the 46 samples^15^ with marked T-cell cytolytic levels, the chi-square test indicated that CEACAM6 high and low levels (by median) is significantly associated with T-cell cytolytic high and low levels (p = 0.03) respectively (Supplementary Table 1). The rate of low T-cell cytolytic activity is higher in the 50% high CEACAM6 expression samples than the 50% low CEACAM6 samples (84.6% vs. 15.4%). The 25% high CEACAM6 samples have a relatively higher rate of low T-cell cytolytic activity than the rest of the samples (p = 0.07). PDA overall is not very immunogenic^15^ but the data shows that the high CEACAM6 are associated with low cytolytic T-cell activity with confidence. These high CEACAM6 low cytolytic TCGA samples constitute both basal and classical subtypes. The T-_REG_ markers (CCR4, CCR5, FOXP3 and IL2RA) are expressed at very low levels in high CEACAM6 samples similar to as expressed in low cytolytic samples^15^.

### CEACAM6 knockout alters the ECM-cell adhesion and immune environment in HPAF-II cells

**Figure 5:**
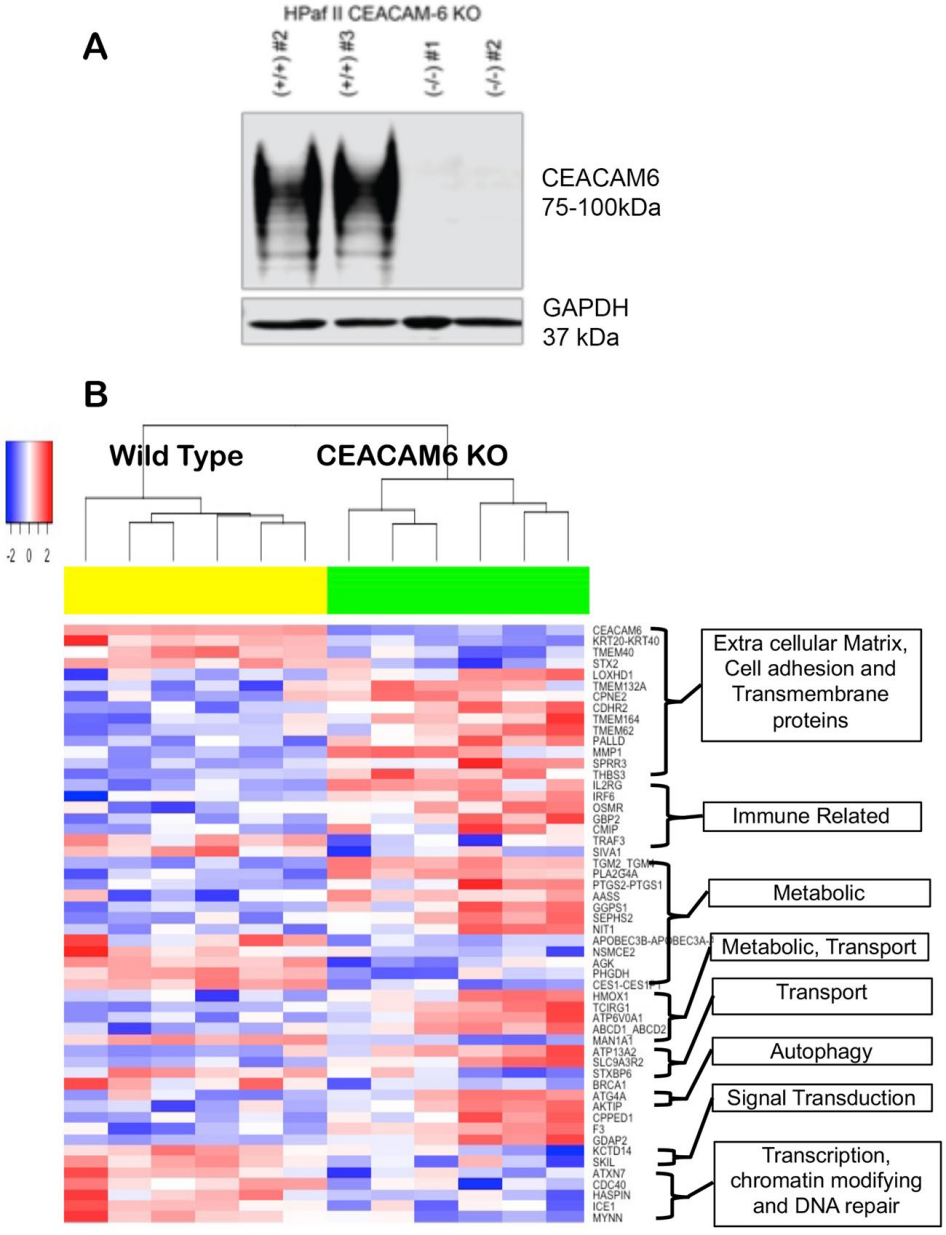
(**A**) Western blotting analysis confirming CEACAM6 KO via CRISPR/cas9 in the HPAF-II PDA cell line and (**B**) Functional association of proteins identified to be altered significantly in CEACAM6 CRISPR/Cas9 Knockout HPAF-II cells.

CEACAM6 expression is highest in the classical PDA cell lines. A CRISPR/Cas9 Knockout (KO) of CEACAM6 in HPAF-II (*KRAS* mutated), a classical type PDA cell line was performed. Western blotting confirmed absence of CEACAM6 in the two KO HPAF-II cells (Fig. 5A). Quantitative proteomics was conducted to evaluate changes in the global HPAF-II cell proteome associated with deletion of CEACAM6, and two separate cultures of CRISPR wild type (WT) clones were profiled against two separate cultures of CRISPR CEACAM6 KO clones, each with three biological replicates. 7698 total proteins were identified across a total of 6 fractions analyzed for the 12 biological samples as described. We found 138 proteins differentially significant (p-value < 0.001) between the WT and CEACAM6 KO HPAF-II samples (Supplementary Fig. 3A, Supplementary Table 2), where 56 proteins had >2-fold differences and the rest have single fold changes.

We identified several ECM and cell adhesion proteins (THBS3, SPRR3, MMP1, PALLD, TMEM62, TMEM164, TMEM132A, CDHR2, ANXA3) being up-regulated (Fig. 5B) and (KRT20 and TMEM40) down-regulated in CEACAM6 KO HPAF-II cells. Several unique proteins are up-regulated in KO cells: *thrombospondin 3* (THBS3) a pentamer held together by inter-chain disulfide bonds is an adhesive glycoprotein that mediates cell-cell and cell-ECM interactions thought to enhance heparin binding^20^ in the TME; *small proline‐rich repeat proteins* (SPRR3) are members of the epidermal differentiation complex (EDC) whose regulation is dependent on integrin α1β1/collagen interaction in response to biomechanical stress^21^, are localized to mitochondria and interacts with Bcl2. Enhanced expression of SPRR3 sensitizes cancer cells to loss of the mitochondrial outer membrane potential (MOMP) that leads to DNA damage-induced apoptosis^22^. These data are consistent with our CEACAM6 KO studies that show reduced MOMP (see below); and increased *palladin*, an actin binding protein that mediates differentiation of cancer associated fibroblast (CAF). It has been demonstrated in PDA cells lacking *KRAS* mutation by gene editing, that they have a slower proliferation but promote gene expression changes that increase metastasis^23^.

In the CEACAM6 HPAF-II KO cells several proteins are altered that are involved in the immune response (IL2RG, IRF6, OSMR, NIT1, CMIP and NFAT2) (Fig. 5B). IL-2RG (CD132), is the highest expressed protein in CEACAM6 KO cells. It is a cytokine receptor subunit common to six IL-receptors (e.g. IL-2, IL-4, IL-7, IL-9, IL-15 and IL-21) that directs growth and maturation of T-cells and NK-cells^24^. In human pancreatic intraepithelial neoplasia-3 (PanIN-3), *IL2RG* was shown to be highly expressed. Knockout by CRISPR/Cas9 of *IL2RG* of bkpc58 cells derived from KPC mice attenuated tumor growth in an orthotopic mouse model of PDA via the Jak3 pathway^25^. However, KPC mice do not express CEACAM6 and a functional correlation to IL-2RG cannot be rationalized. Interferon regulatory factor 6 (IRF6) is a transcription factor but unlike other IRF family members, it is not involved in interferon (IFN) gene expression and is up-regulated in the HPAF-II CEACAM6 KO cells. Instead, IRF6 is involved in cell adhesion, motility, control of epidermal precursor proliferation and acts as a tumor suppressor for invasiveness and proliferation^26^.

### Gene editing of CEACAM6 in HPAF-II cells increases catabolism, transmembrane transport, and decreases DNA repair processes

Gene set analysis revealed representation of catabolic processes, biochemical homeostasis, regulation of cell proliferation, transmembrane transport and inflammatory response in the KO cells. Alterations to metabolic processes are evident by significant increases in TGM2, PLA2G4A, members of ATPase H^+^-transporting unit and several mitochondrial proteins in KO cells. The KO cells appears to be striving for more ATP utilization, as ATPase enzymes were up-regulated. Increase in breakdown of substances and carbon compounds and liberation of energy indicates increased catabolic processes (ATG4A, PLA2G4A, MMP1, HMOX1, AASS, ABCD1, AMPD3, STBD1, CTBS, PLBD2 and GLS) (Supplementary Fig. 3B). ATPase activity coupled to movement of substances is also up-regulated in KO cells (ATP6V0A1, ATP6V0D1, TC1RG1, ATP13A2, ABCC10) (Supplementary Fig. 3B) and results in increased transmembrane transporter activity. We observed a decrease in RNA binding, chromatin organization/modification and cell cycle DNA repair proteins (INO80C, MTA1, MSL1, SETX, HSPA2, CHD7, DDB2, NUF2, BRCA1, NSMCE2, ATXN7) in KO cells (Supplementary Fig. 3B). Some of these decreased proteins are also involved in metal-ion binding functions. Interestingly, APOBEC3B (A3B) a DNA cytosine deaminase, is a source of genomic DNA mutations that contributes to cancer progression and metastasis is significantly down-regulated in CEACAM6 KO cells. Proteins involved in response to oxidative and cellular stress such as ATG4A, MAP4K4 and HSP70 family members are increased in the KO cells.

### CEACAM6 loss alters mitochondrial functions in PDA

**Figure 6:**
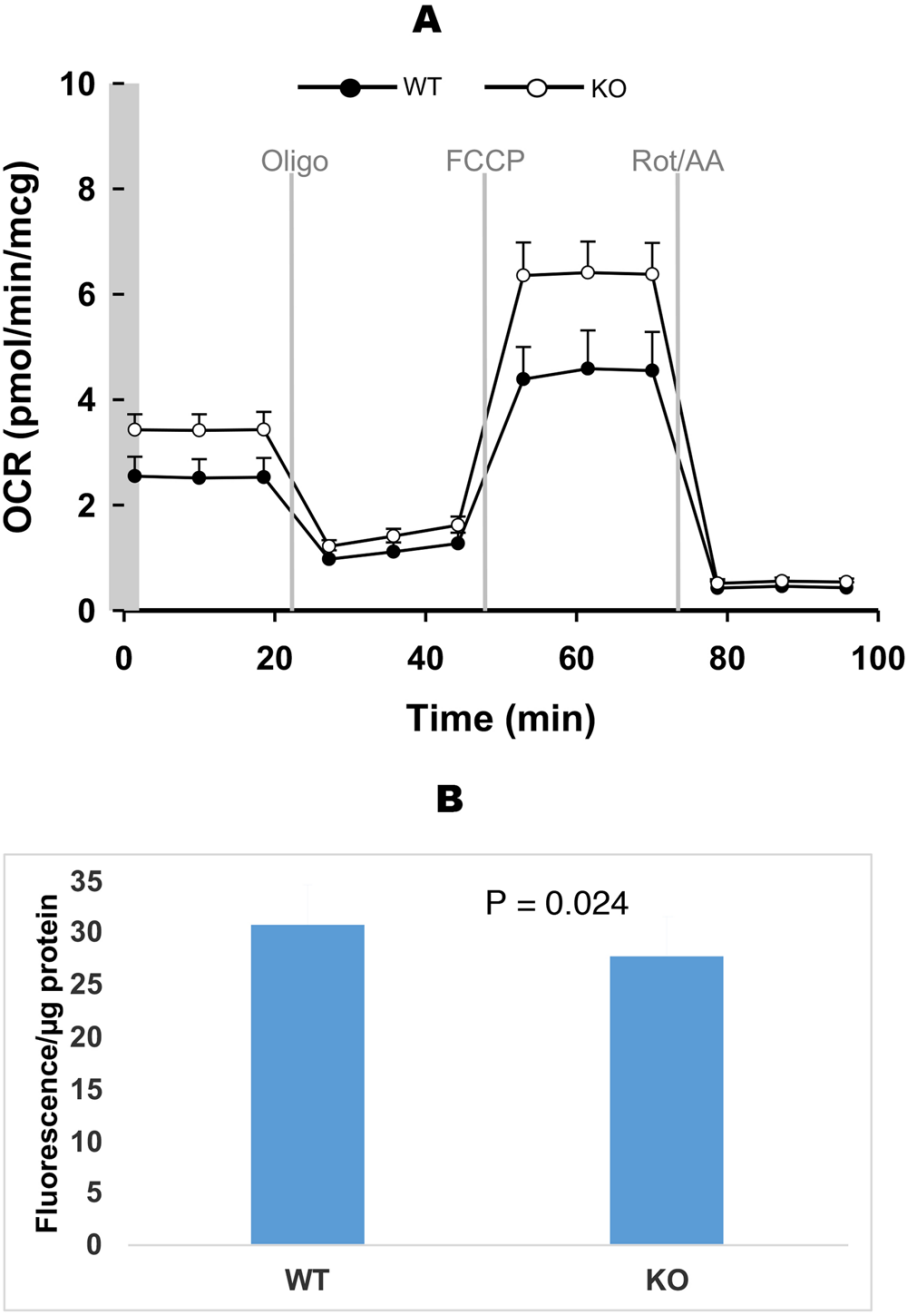
(**A**) Mitochondrial bioenergetics detected as oxygen consumption rate (OCR) of HPAF-II CEACAM6 +/+ (black circle) and HPAF2 CEACAM6−/− (open circle) cells. Mitochondrial function parameters were calculated based on the OCR profiles following the recommended guidelines. (**B**) Mitochondrial membrane potential as measured by TMRM.

We next investigated mitochondrial function in WT versus KO HPAF-II PC cells using the Seahorse Extracellular Flux Analyzer technology. CEACAM6 HPAF-II KO cells show higher basal respiration (26% higher), and maximum respiratory capacity (29% higher) compared to WT cells (Fig. 6A). These findings are in line with the gene editing results, where we observe increased catabolism and ATPase activity. The high energetic demand in CEACAM6 HPAF-II KO cells is supported by increased mitochondrial function. Proteomics of the KO cells demonstrated increased catabolic processes in an attempt to increase ATP utilization, by increased ATPase enzymes. CEACAM6 KO cells have a significant effect on mitochondrial energy metabolism. The mitochondrial outer membrane potential (MOMP) of the KO cells was lower reflective of high energy demand and consequently higher ATP production rates (Fig. 6B).

### Expression changes in CEACAM6 KO cells in comparison to human PDA

Analysis of independent GEO datasets for differential expression between normal pancreas and PDA found 2989 genes commonly up-regulated in tumors across all the datasets (Supplementary Fig. 4A). We compared these gene changes to CEACAM6 KO protein changes. For some of the genes we see reversal of expression in KO cells compared to PDA samples. *KRT20* is high in tumors and is significantly down in KO cells. TCIRG1 is low in tumors and is high in KO cells. ATG4A, ATP13A2, AASS SERPINH1, SKIL, CDHR2 and TRAF3 all change in expression upon KO. There were also genes that are of insignificant expression in tumor cells that increase in expression in KO cells, e.g. THBS3, IL2RG, SPRR3, TGM2, HMOX1 and TMEM62 or are lower in expression in tumor cells and significantly decreased in KO cells such as MAN1A1, CES1, STCBP6, SIVA1 and TMEM40.

Correlation matrix assessment for CEACAM6 across tumor and normal samples identified 667 genes positively correlating and 171 genes negatively correlating with CEACAM6, with a correlation score of >0.4 and P value < 0.05. Gene set enrichment analysis shows CEACAM6 correlates significantly with ECM-cell adhesion proteins, collagens, integrins, extra-cellular protein-receptor interactions, and basement membrane proteins involved with ECM organization (Supplementary Fig. 4B). These genes are enriched in signatures for epithelial-to-mesenchymal transition and *KRAS* signaling. In addition, genes positively correlated with CAECAM6, were also enriched with key immunologic signatures involved in differentiation and proliferation of T-cells. These immunologic signatures are derived from stimulation or perturbation of CD4+ and CD8+ T-cells by over-expressing T-_REG_ markers and/or elaboration of cytokines, reduction of certain cytokine-activated proteins and/or T-cell immune environment activation upon infection. Further, CEACAM6 is high in activated stroma and a pairwise CEACAM6 correlation found 745 genes positively correlated with CEACAM6 with a correlation score of >0.4 and P value < 0.0. Gene set analyses revealed that similar functional processes are enriched in the activated stroma of PDA (Supplementary Fig. 4C,D).

### CEACAM6 knockdown suppresses tumor growth in SCID mice

**Figure 7:**
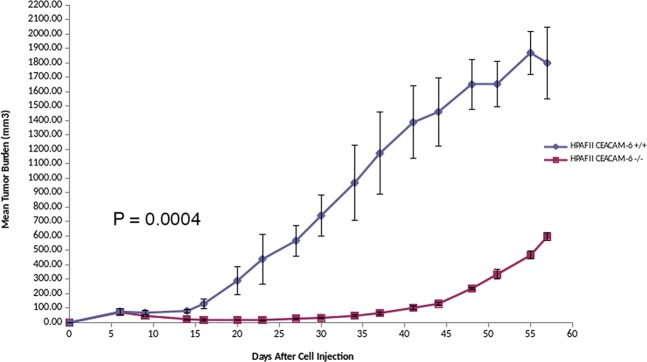
HPAF-II CEACAM6−/− [KO, RED] (CRISPR/Cas9 gene edited) Vs. HPAF-II CEACAM6 +/+ [WT, BLUE] cells subcutaneously grown in SCID mice (n = 4 in each cohort). Tumor growth suppression is >95% as observed at day 30 in the CEACAM6 knockout cells. At day 40–45 tumors begin to grow albeit at a slower growth rate compared to HPAF-II WT cells with a final tumor growth suppression of ~66% at day 57.

HPAF-II cells with CEACAM6 knockout grow ~40% slower than the WT cell line and have a significantly diminished proliferation and ability to form subcutaneous tumors in SCID mice. The delta for tumor growth suppression is >95% at day 30 (Fig. 7). However, at day 40–45 tumors begin to grow albeit at a significantly reduced growth rate, with ~66% growth suppression at day 57. This is in contrast to faster tumor growth of *KRAS* knockout mutant of ~35% growth suppression vs. *KRAS* mutant cells^27^. Supplementary Figure 6A shows HPAFII subcutaneous tumor from CEACAM6 −/− and CEACAM6 +/+ mice at the end of the study. Kaplan-Meier survival curves show a difference in overall survival (Supplementary Fig. 6B). Mice were weighed before the beginning of the experiment and once/week thereafter and mice body weight in CEACAM6−/− vs CEACAM6 +/+ showed no reduction in weight. (Supplementary Fig. 6C). Although *KRAS* mutations are a major driver of PDA, CEACAM6 KO in KRAS mutant PDA cells severely compromised tumor growth. Expression profiles of PDA cells of mutant *KRAS* knockout showed a role for metastasis suppression related genes. Our proteomic data on HPAF-II cells ± CEACAM6 showed an anti-metastasis profile likely due to alteration to multiple cellular processes. This study validates CEACAM6 as a potential therapeutic target in pancreatic cancer. We have developed a therapeutic humanized monoclonal antibody to CEACAM6, which showed promising anti-tumor activity in a mouse xenograft model^10^.

## Discussion

CEACAM6, an oncotarget, has multi-faceted roles that include anoikis drug resistance and immune suppression^28^ within the TME in cancer progression and metastasis^29^. It is an attractive therapeutic target and if disrupted may provide a multi-targeted attack on classical stroma rich PDA^10^. We demonstrated that CEACAM6 levels are significantly low in normal pancreas and normal other organs compared to either from primary and/or at metastatic PDA (classical and basal). In addition, CEACAM6 over-expression correlated with classical activated enriched for activated stroma represented in metastatic classical and basal subtypes. Elevated CEACAM6 expression negatively influences overall survival in PDA patients. The 50% probability of survival with high CECAM6 levels is ~500 days versus not reached (NR) for low CEACAM6 expressers (p = 0.02) with no prior systemic therapy. The Kaplan-Meier survival curves for classical subtype PDA stratified by low and high CEACAM6 expression at 50% had a survival of ~800 days versus ~400 days (p = 0.01) respectively at Whipple’s procedure. In addition, *KRAS* mutated tumors had a higher tendency to over-express CEACAM6 versus WT *KRAS* tumors. *KRAS* WT PDA patients with high CEACAM6 expression have a poor overall survival. CEACAM6 over-expression mirrors the prognostic significance of oncogenic *KRAS* in >90% PDA patients.

Members of the CEA family are expressed in higher vertebrates and are known to have tumor-associated immune functions in T-cells, NK-cells and neutrophils^30^. In inflammatory colon disease and multiple myeloma, CEACAM6 is known to activate a subset of immune suppressive CD8+ T-_REG_ cells^28^. We show that high CEACAM6 expression is associated with immune suppression in classical subtypes exemplified by a low level of cytolytic T-cell activity particularly in the activated stroma subtype PDA. Proteomics of CEACAM6 KO HPAF-II cells provide evidence that IL-2RG (CD132), a cytokine receptor subunit is up-regulated and capable of acting via IL-receptors to direct growth and maturation of T-cells and NK-cells which are generally absent in PDA.

CEACAM6 KO impacts several hallmarks of cancer including ECM-cell adhesion, transmembrane proteins, metabolism and transport, autophagy, DNA repair, chromatin modifications and signal transduction. We identified cell-adhesion/ECM genes that are down-regulated including KRT20 (cytokeratin 20) which is over-expressed in many epithelial carcinomas. Also, the transmembrane protein 40 (TMEM40), an oncogene known to play a key role in proliferation and anti-apoptosis via the p53 signaling pathway in bladder cancer^31^ is down-regulated in the KO cells. Of the up-regulated cell-adhesion/ECM proteins SPRR3 stands out, as its regulation is dependent on α1β1/collagen interaction that contributes to mechano-reciprocity^21^ that may be important to reshaping the fibrotic reaction in PDA. In CEACAM6 KO cells changes in ECM proteins do not promote increased migratory behavior as seen in the mouse xenograft model. Since mitochondria play a key role in the fibrotic processes in PDA, high expression of SPRR3 may sensitize HPAF-II cells lacking CEACAM6 to DNA damage-induced apoptosis via the loss of mitochondrial outer membrane potential (MOMP).

ATG4A, a cysteine protease is up-regulated in CEACAM6 KO HPAF-II cells which is required for cytoplasm to vacuole transport (Cvt) and autophagy that can be targeted with tioconazole^32^. There is an increased catabolism by breakdown of substances including carbon compounds. Glutaminase (GLS), an amidohydrolase converts glutamine to glutamate is down-regulated in KO PDA cells. It is known that many cancers are addicted to glutamine via the Warburg effect and are dependent on GLS for survival^33^. KO cells have up-regulated transglutaminase2 (TGM2) a multifunctional enzyme that catalyzes the covalent cross-linking of proteins in a calcium-dependent manner by utilizing glutamine and lysine^34^. This is most likely a direct effect of increased glutamine requirements due to down-regulation of GLS. In addition, TGM2 participates in regulated cell death, when its transamidating activity is fully operational but plays a protective role when the transamidating activity is non-operational^35^. When TGM2 is secreted into the extracellular space, it has an adhesive role of stabilizing the ECM^36^.

Mitochondria generate oncometabolites, ROS, and an altered MOMP that allows tumor cells to escape regulated cell death and compromise immune functions. Oncogenic *KRAS* in PDA can enhance mitochondrial succinate and fumarate production but also increases the resistance of the mitochondrial pool to MOMP by relying on oxidative phosphorylation for energy production, promoting its oncogenic program^37^. HPAF-II CEACAM6 KO cells have a significantly reduced MOMP but higher ATP production which is a consequence of activation of mitochondrial oxidative phosphorylation compared to WT cells. The KO cells demonstrate a significant increase in oxygen consumption rate (OCR) with a dependence on glutamine-dependent reduction of carbon. In the KO cells this is achieved by down-regulation of GLS and up-regulation of TMG2, which in addition impacts the reshaping of the fibrotic stroma by modulating the ECM. Further, the membrane passive proton conductance is also significantly affected in the CEACAM6 KO cells compromising the coupling efficiency between electrons transfer and proton extrusion via proton pumps.

Elevated expression of APOBEC3B (A3B) correlates with kataegic patterns of localized hypermutation in human tumors. In CEACAM6 KO cells the DNA damage response (DDR) is suppressed and there is also a significant decrease in the expression A3B, an intrinsic mutagen that contributes to cancer progression and metastasis^38^. Continued expression of A3B is observed in p53-defective cells and are hypersensitive to DDR inhibitors (e.g. ATR, CHEK1, CHEK2, PARP, WEE1 inhibitors). Disruption of CEACAM6 may sensitize cells with *TP53* mutations to the aberrant DDR signatures elaborated in some PDAs.

Finally, HPAF-II cells with loss of CEACAM6 demonstrated a significantly diminished ability to form subcutaneous tumors in SCID mice compared to wild type cells. At day 30, tumor growth suppression was >95% in the CEACAM6−/− vs. CEACAM6 +/+ tumors. This level of tumor suppression observed is significantly higher compared to mutant *KRAS* knockout PDA cell growth of subcutaneous tumors in mice^27^, implicating a relationship of *CEACAM6* to *KRAS* in PDA that needs further investigation. Previously we showed that a recombinant humanized monoclonal antibody (scFv) to CEACAM6 induced apoptosis by PARP cleavage in cell culture, inhibited of cell proliferation via disruption of anchorage independence and anti-tumor activity in a mouse xenograft model of PDA alone and synergistically with gemcitabine^10^. We have now generated an anti-CEACAM6 scFv-Fc (IgG4) and will be evaluating anti-tumor activity in PDA mouse models.

In summary, CEACAM6 is a key oncogenic driver affecting several key hallmarks that provide a survival advantage to PDA. Importantly, CEACAM6 plays important roles in modulating ECM-cell adhesion leading to anoikis chemo-resistance, immune suppression, and mitochondrial metabolic defects in PDA. Disrupting CEACAM6 provides a therapeutic approach to target multiple abnormalities intrinsic to PDA and provide novel treatment regimens to be evaluated pre-clinically and in the clinic. Deconvolution of the myriad of intrinsic and extrinsic tumor associated factors^39,40^ to CEACAM6 regulation requires further investigation.

## Material and Methods

### RNA-Seq and array data processing

PDA samples from The Cancer Genome Atlas (TCGA) were downloaded from Genomic Data Common Website (GDC at https://gdc.cancer.gov/). A total of 177 tumor samples with RNA-seq assay were obtained from TCGA. GDC data transfer tool client and GDC API was used to download all the RNA-seq raw counts data, metadata and available clinical data. The array data on PDA patients and the clinical data from the Australian Pancreatic Cancer Genome Initiative and the International Cancer Genome Consortium (ICGC) was downloaded from http://icgc.org. Data was analyzed using R (v3.4.3). Raw HTSEQ counts data was normalized using Variance Stabilizing Transformation (VST) method^41^ and voom^42^ and the data was processed further. PDA gene Expression datasets were downloaded from GEO. Normalized log transformed array data were analyzed further. Differential analysis between groups was done using the DESeq2 and Limma^43^. Arrays GSE16515, GSE15471 and GSE17891 were annotated using Bioconductor annotation package hgu133plus2.db. GSE71729 probes were annotated using Agilent Human Genome Microarray annotation. MicroRNA, probes without symbols, and probes with multiple symbols were removed from the data. Multiple probes matching to the same gene symbol were collapsed by mean probe expression.

### Survival analysis of samples with over-expressed CEACAM6

Gene expression profiles were divided into higher and lower categories based on CEACAM6 normalized expression. A median cutoff based on expression was taken to divide the samples into two categories. The long-term overall survival was analyzed by utilizing the Kaplan-Meier survival plot. Analysis of survival time was performed using R statistical software and the survival package. Log-rank test was applied to assess the difference between the survival cohorts. The survdiff function under rms library in R was used to perform the log-rank test. The hazard ratio was calculated from cox proportional hazard model using coxph function under the survival library in R.

### Functional analysis

Differentially expressed genes in PDA samples and CEACAM6 knockout samples were analyzed for MsigDB gene sets using GSEA (http://software.broadinstitute.org/gsea/msigdb/index.jsp). Differential genes between normal and tumor, within tumor between activated and normal stroma and proteins upon CEACAM6 knock out in HPAF II cells were evaluated for over representation or change in biologically related functional gene sets.

### Association analysis

The Chi-square test was used to evaluate the associations for categorical data and Fishers exacts for the association between dichotomous outcomes and CEACAM6. Two-sided p values of less than 0.05 were regarded as significant.

### Correlations

A correlation matrix was built for CEACAM6 for independent datasets (GSE15471, GSE16515, GSE71729) of normal and tumor samples. The Pearson’s correlation method was used. Positive and negative correlations with CEACAM6 with r >0.4 and P < 0.05 were investigated further. Similarly, a correlation matrix was built for CEACAM6 across activated stroma and normal stroma subtypes of PDA tumors.

### CRISPR/Cas9 editing of CEACAM6 in PDA cell lines

PDA cell line HPAF-II was obtained from ATCC. Cell line authentication was performed using Promega PowerPlex16HS Assay and tested for mycoplasma contamination using MycoAlert Mycoplasma Detection Kit (Lonza). HPAF-II cell line was cultured in DMEM medium supplemented with 10% fetal bovine serum at 37 °C in a humidified atmosphere containing 5% CO2. Homozygous knockout (KO) of PDA cell line (HPAF-II) for CEACAM6 were generated using CRISPR/Cas9 technologies in the UACC Genome Editing Facility. Double strand breaks were produced on either side of the entire CEACAM6 transcription unit at predicted sites 19:41,746,767 and 19:41,777,352 with guides corresponding to the sequences 5′-TCTTAGACTCGCCCGCATCT-3′ and 5′-AGGTGTTTTGGACACTACGC-3′. Parental PDA HPAF-II (*KRAS* mutated) cells were transfected with Cas9 protein, crRNAs, and tracrRNA (Integrated DNA Technologies) using the Lipofectamine RNAiMAX reagent (Thermo Fisher Scientific). Tw o days after transfection, cutting efficiency was estimated based on DNA prepared from a portion of the transfected cell population using a T7 endonuclease assay (New England BioLabs) employing PCR primers flanking the predicted ligation-junction product (5′-GCCATGAGATGTGTGGAGAAAGA-3′ and 5′-ATTGCTAGTTGGCTCTAACCT-3′). Single cells were deposited in ten 96-well plates and colonies expanded and screened by PCR and agarose gel electrophoresis. Clones that were negative for two fragments internal to the targeted deletion (fragment 1, 5′-GGCTTTAGCCCTGGATGTGT-3′ and 5′-CAGGAGACCCTAGCCAGTCT-3′; fragment 2, 5′-TAAGGCTCAGGGTTCACATTTTCT-3′ and 5′-AGAGTCTGCAGAGGTGAATTGG-3′) but positive for the ligation-junction fragment were potentially homozygous for the deletion. Absence of CEACAM6 protein was confirmed by Western blotting, performed as described in^44^, using anti-Ceacam-6 (SC# 59899) antibody purchased from Santa Cruz Biotechnology (Dallas, TX) and anti-GAPDH (CST# 2118) antibody purchased from Cell Signaling Technology (Danvers, MA) (Supplementary Fig. 5).

### Quantitative proteomics

Forty (40) μg of the HPAF-II cell lysate supernatant was separated on a 10% SDS-PAGE gel and stained with Bio-Safe Coomassie G-250 Stain. Each lane of the SDS-PAGE gel was cut into six slices and the samples were subjected to tryptic digestion and desalting as previously described^45^. The dried peptides were resuspended in 6 μl of 0.1% FA (v/v), sonicated for 1 min and 2.5 μl of the final sample was analyzed by mass spectrometry. Progenesis QI for proteomics software (version 2.4, Nonlinear Dynamics Ltd., Newcastle upon Tyne, UK) was used to perform ion-intensity based label-free quantification as previously described^46^. The peak list data was searched against the human SwissProt_2018_01 database, (21518 entries) using Mascot (Matrix Science, London, UK; version 2.4). Two separate cultures of CRISPR WT clones were profiled against two separate cultures of CRISPR CEACAM6 KO clones, each with three biological replicates. Global protein expression changes were compared between WT (control) versus KO cells using protein abundance values. First, a t-test statistic to find significantly different proteins was applied. Secondly, a data reduction strategy using exclusion criteria to only those proteins that were not significantly different within their WT clones versus KO clones, but significantly different between any one WT clone versus both KO clones and vice versa between any KO clone versus both WT clones were chosen.

### Measurement of mitochondrial bioenergetics

CEACAM6 WT and KO HPAF-II cells were seeded in Seahorse cell culture microplates (Seahorse Bioscience #100777-004, 24-well plates), at 40,000 cells/well. Bioenergetic assays (oxygen consumption rates, or OCR) followed Seahorse Bioscience protocols using a machine XFe 24 Extracellular Flux Analyzer (Seahorse Bioscience). To assay oxidation of carbohydrate in the form of added glucose in intact cells, XF Base Medium (Seahorse Bioscience #102353-100) with 10 mM glucose, 2.0 mM glutamine and 1.0 mM pyruvate was used. Other test components included 1.0 µM oligomycin, 1.0 µM FCCP, and 2.0/2.0 µM rotenone/antimycin A. Respiration (OCR) in each well was normalized to cell lysate protein concentration.

### Mitochondrial membrane potential (MMP)

CEACAM6 WT and KO HPAF-II cells were seeded at a density of 40,000 cells in a 96-well clear bottom plate (VWR #29444-008) and cultured for two days. Cell growth medium was removed and 100 μl of growth medium with 50 nM TMRM (ThermoFisher #T668) was added to the cells. The cells were stained for 30 min in cell incubator at 37 °C 5% CO_2_. After staining, cells were washed once with PBS and then added with 100 μl of PBS for measurement. The fluorescence signals (Ex 548/Em 574) were measured using a reader BioTek Synergy H1.

### Subcutaneous tumor growth in mice

All animal experiments were conducted in accordance with the guidelines of the Institutional Animal Care and Use Committee (IACUC) of the University of Arizona under IACUC protocol # 07–029 approved 01/22/2019. Animals were housed at the University of Arizona’s Animal Care Facility in accordance with the University of Arizona Institutional Animal Care and Use Committee guidelines. We evaluated the growth of HPAF-II CEACAM6−/− Vs. CEACAM6 +/+ cells in SCID mice with tumors growing subcutaneously. Growth constant, k, was calculated for tumors growing in mice using the exponential growth formula y(t) = ae^(kt) for WT vs KO HPAF-II cell lines. For the WT cell line, k ~ 0.058 and for the KO line, k~0.034. The CEACAM6 KO HPAF-II cell line grows ~40% slower than the WT cell line. Four mice per cohort were inoculated with 10 × 10^6^ cells using a subcutaneous injection in sterile saline. Tumor growth was measured twice/week with calipers. The tumor volume was estimated using the formula [(width)^2^ × length]/2). All harvested tumors at the end of study were formalin-fixed paraffin-embedded (FFPE) for hematoxylin and eosin staining for size, weight and histologic assessment. When tumors reach 2000 mm^3^ they were sacrificed and tumor was collected for further studies. Kaplan-Meier survival was calculated. Mice were weighed before the beginning of the experiment and once/week thereafter to check for signs of illness.

### Statistics

Bioinformatics Analysis of clinical data and experimental results from the expression data was carried out in the R (v 3.4.3). No statistical method was used to predetermine sample size of published experimental data for CEACAM6 analysis. Unpaired t-test was used to compute statistical significance. All plots and heat maps were generated using the gplots library in R.

### Ethics approval and consent to participate

All animal experiments were conducted in accordance with the guidelines of the Institutional Animal Care and Use Committee (IACUC) of the University of Arizona, Tucson, USA under IACUC protocol # 07–029 approved 01/22/19 (Also indicated in Materials and Methods Section).

## Supplementary information

Supplementary Information

## Data Availability

Publicly available data was used for the study.
