# Supplementary material for: Carcinoembryonic antigen cell adhesion molecule 6 (CEACAM6) in Pancreatic Ductal Adenocarcinoma (PDA): An integrative analysis of a novel therapeutic target
Source: Sci Rep. 2019 Dec 4;9:18347. doi: 10.1038/s41598-019-54545-9 (PMC6893022; doi:10.1038/s41598-019-54545-9)
Supplement: Supplementary file 1 — Supplementary Information [file 41598_2019_54545_MOESM1_ESM.pdf]

Online Supplemental Material

Title Page:

Carcinoembryonic antigen cell adhesion molecule 6 (*CEACAM6*) in Pancreatic Ductal Adenocarcinoma (PDA): ***An integrative analysis of a novel therapeutic target***

Authors and affiliations:

Ritu Pandey[1,2,*], Muhan Zhou[1], Shariful Islam[1], Baowei Chen[1], Natalie K Barker[3], Paul Langlais[3], Anup Srivastava[3], Moulun Luo[3], Laurence S Cooke[1], Eric Weterings[1,3,4], Daruka Mahadevan[1,3,*]

1. University of Arizona Cancer Center, University of Arizona, Tucson, USA

2. Department of Cellular and Molecular Medicine, University of Arizona, Tucson, USA

3. Department of Medicine, College of Medicine, University of Arizona, Tucson, USA

4. Department of Radiation Oncology, College of Medicine, University of Arizona, Tucson, USA

*** CO-CORRESPONDING AUTHORS:**

Daruka Mahadevan, MD, PhD - Email: dmahadevan@uacc.arizona.edu, 1515 N Campbell Avenue, Room 1905, University of Arizona Cancer Center, Tucson, AZ, 85724

Ritu Pandey, PhD - Email: ritu@email.arizona.edu, 1515 N Campbell Avenue, Room 1932, University of Arizona Cancer Center, Tucson, AZ, 85724

Competing Interests

The author(s) declare no competing interests.

Online Supplemental Material

Supplementary Figure 1

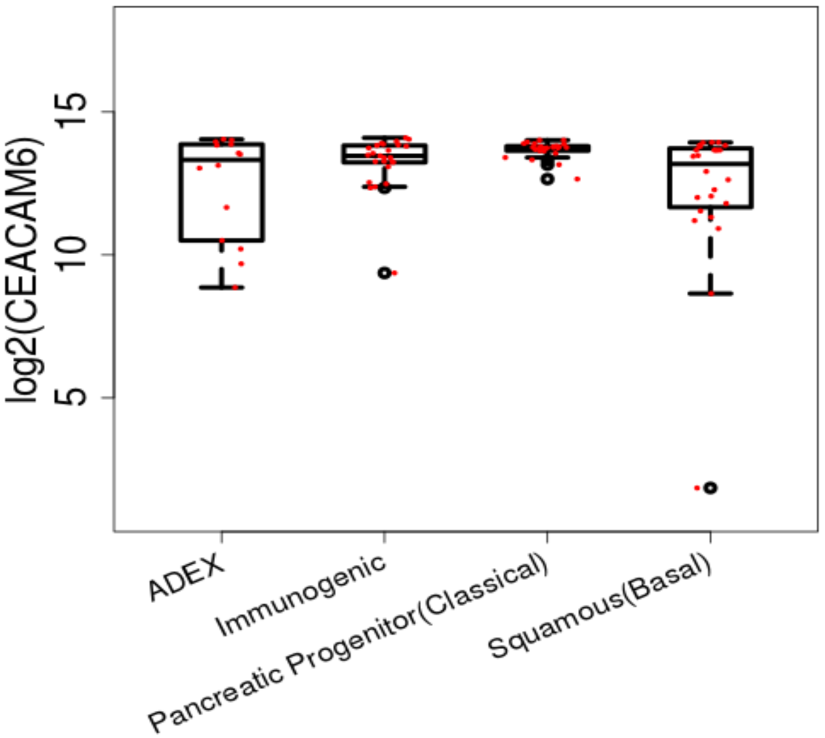

Supplementary Figure 1: Box plots showing expression of CAECAM6 across different tumor types in the ICGC data.

Supplementary Figure 2A

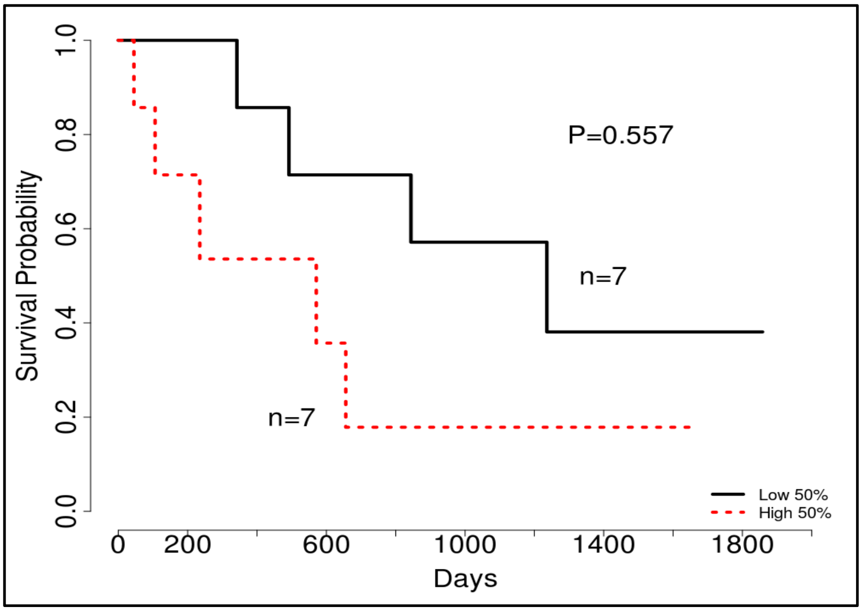

Supplementary Figure 2: A) Kaplane Meier survival analysis of classical tumor types stratified by median expression of CEACAM6 in data from GSE17891. Plot shows the different distribution of survival between two groups.

Online Supplemental Material

Supplementary Figure 2B

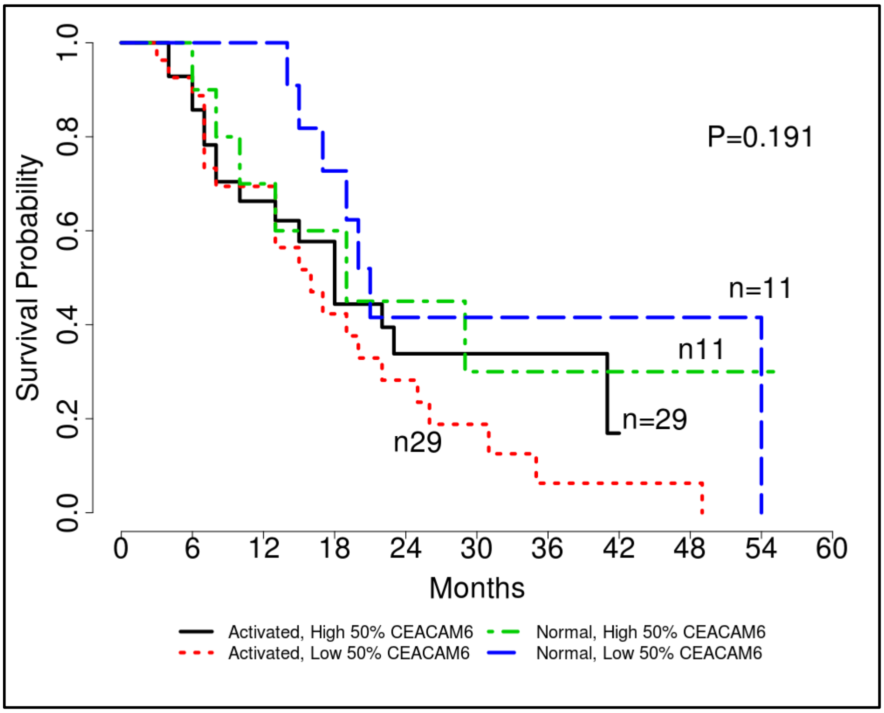

Supplementary Figure 2C

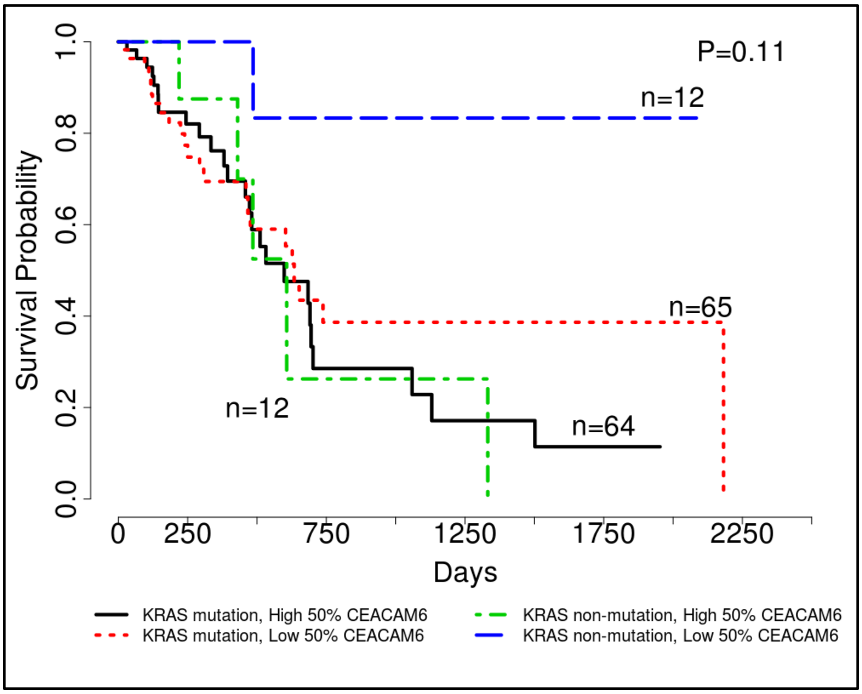

Supplementary Figure 2: B) Kaplan-Meier survival analysis of activated stroma and normal stroma stratified by CEACAM6 expression. Plot shows survival distribution across four groups. C) Kaplan-Meier survival analysis of PDA patients in presence and absence of KRAS mutations.

Online Supplemental Material

Supplementary Figure 3 A

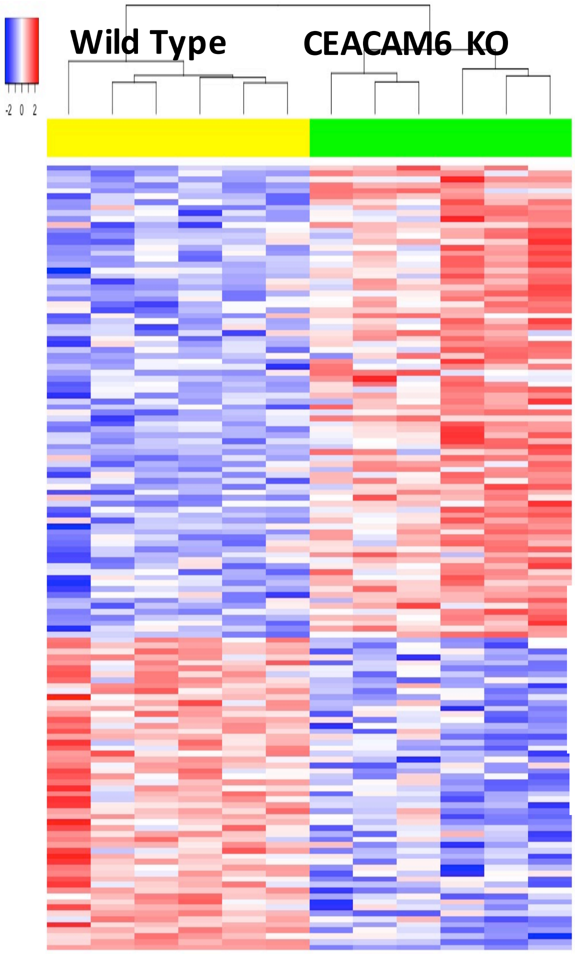

Supplementary Figure 3 B

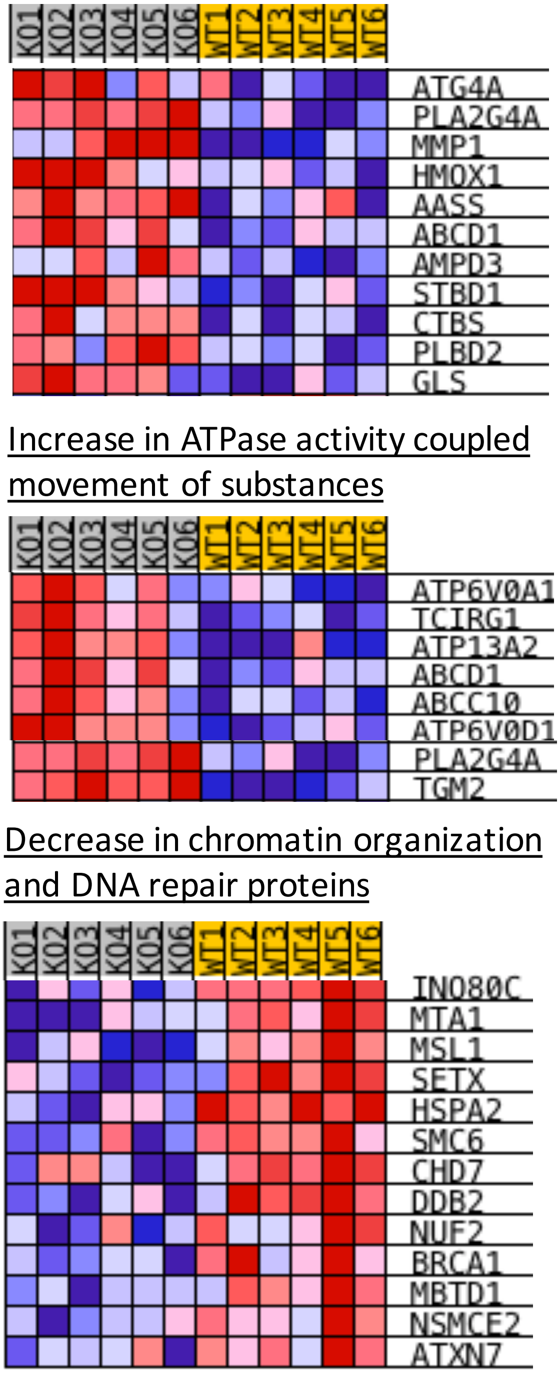

Supplementary Figure 3: A) Unsupervised clustering of 138 proteins from HPAFII wild type and CEACAM6 KO clones and B) Key processes affected by CECAM6 KO in HPAFII cells

Online Supplemental Material

Supplementary Figure 4 A

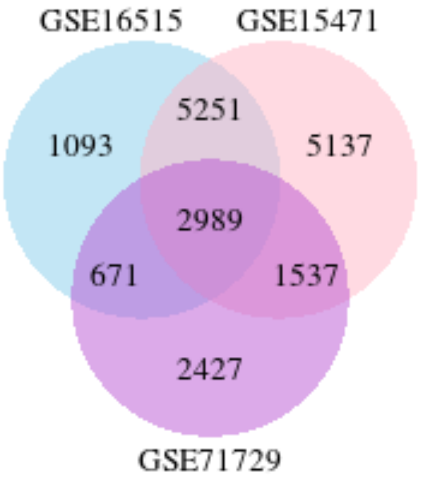

Supplementary Figure 4 B

MSigDB GENESET	SIGNATURES	SIZE	ES	NES	NOM p-val	FDR q-val
GO	EXTRACELLULAR Matrix Components	44	0.400	1.500	0.043	0.734
GO	EXTRACELLULAR Matrix Components	44	0.710	2.620	0.000	0.000
GO	EXTRACELLULAR Matrix Components	44	0.410	1.670	0.004	0.420
HALLMARK	EPITHELIAL MESENCHYMAL TRANSITION	37	0.490	1.930	0.001	0.041
HALLMARK	EPITHELIAL MESENCHYMAL TRANSITION	37	0.790	2.770	0.000	0.000
HALLMARK	EPITHELIAL MESENCHYMAL TRANSITION	37	0.340	1.340	0.122	0.684
HALLMARK	KRAS SIGNALING UP	28	0.410	1.510	0.034	0.738
HALLMARK	KRAS SIGNALING UP	28	0.610	2.060	0.000	0.000
HALLMARK	KRAS SIGNALING UP	28	0.510	1.910	0.001	0.023
Immunologic Signatures	GSE36888_UNTREATED_VS_IL2_TREATED_TCELL_17H_DN	21	0.579	1.958	0.000	0.465
Immunologic Signatures	GSE36888_UNTREATED_VS_IL2_TREATED_TCELL_17H_DN	21	0.568	2.043	0.000	0.121
Immunologic Signatures	GSE30971_2H_VS_4H_LPS_STIM_MACROPHAGE_WBP7_HET_DN	11	0.658	1.879	0.000	0.764
Immunologic Signatures	GSE30971_2H_VS_4H_LPS_STIM_MACROPHAGE_WBP7_HET_DN	11	0.705	1.899	0.000	0.310
Immunologic Signatures	GSE37416_CTRL_VS_48H_F_TULARENSIS_LVS_NEUTROPHIL_UP	18	0.537	1.770	0.010	0.893
Immunologic Signatures	GSE37416_CTRL_VS_48H_F_TULARENSIS_LVS_NEUTROPHIL_UP	18	0.593	2.032	0.001	0.091
Immunologic Signatures	GSE18281_CORTICAL_VS_MEDULLARY_THYMOCYTE_UP	29	0.470	1.741	0.003	0.937
Immunologic Signatures	GSE18281_CORTICAL_VS_MEDULLARY_THYMOCYTE_UP	29	0.544	1.852	0.001	0.390
Immunologic Signatures	GSE42021_TREG_VS_TCONV_PLN_UP	28	0.542	1.853	0.001	0.408
Immunologic Signatures	GSE42021_TREG_VS_TCONV_PLN_UP	28	0.564	2.105	0.000	0.121
Immunologic Signatures	GSE13485_DAY3_VS_DAY7_YF17D_VACCINE_PBMC_DN	36	0.515	1.840	0.000	0.382
Immunologic Signatures	GSE13485_DAY3_VS_DAY7_YF17D_VACCINE_PBMC_DN	36	0.491	1.923	0.001	0.203
Immunologic Signatures	GSE43955_1H_VS_20H_ACT_CD4_TCELL_WITH_TGFB_IL6_DN	20	0.577	1.794	0.004	0.463
Immunologic Signatures	GSE43955_1H_VS_20H_ACT_CD4_TCELL_WITH_TGFB_IL6_DN	20	0.560	1.942	0.001	0.184
Immunologic Signatures	GSE2706_2H_VS_8H_LPS_STIM_DC_DN	19	0.557	1.904	0.000	0.220
Immunologic Signatures	GSE2706_2H_VS_8H_LPS_STIM_DC_DN	19	0.574	1.779	0.007	0.514

Supplementary Figure 4: A) Venn diagram showing number of differential expressed genes between tumor and normal types across three independent GEO datasets. B) GSEA of Genes that are correlated with CEACAM6 in tumor samples. All three datasets were independently assessed for MSigDB signatures. For immunologic signatures top signatures represented in two independent datasets were chosen.

Online Supplemental Material

Supplementary Figure 4C

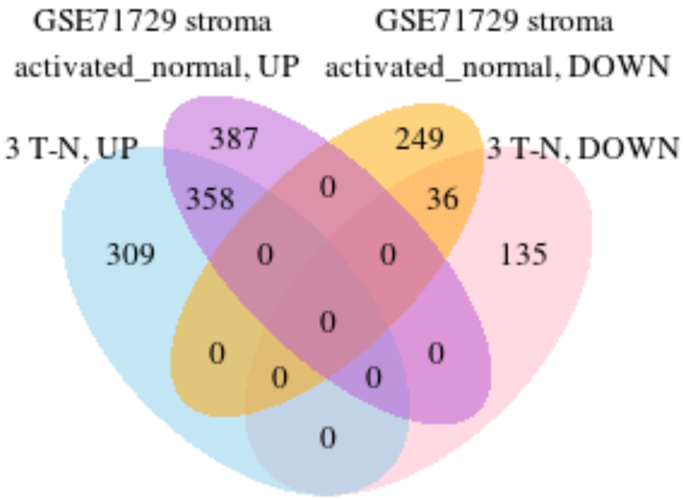

Supplementary Figure 4D

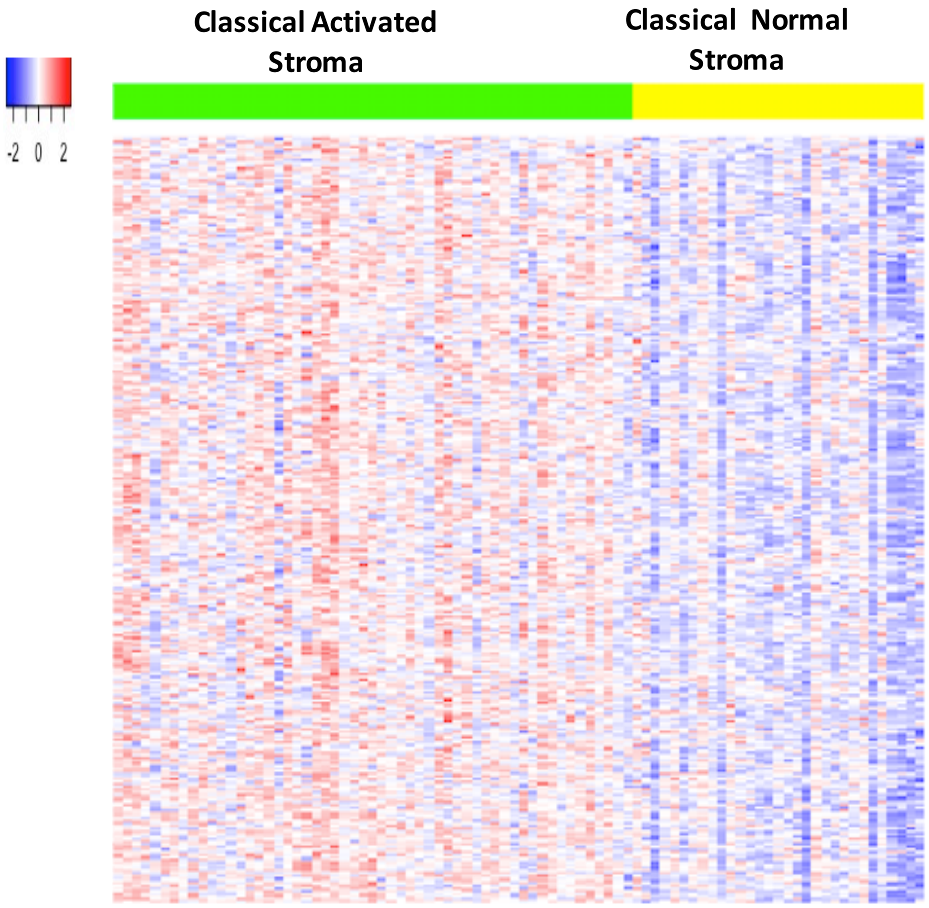

Supplementary Figure 4: C) Venn diagram showing positive correlation of CEACAM6 with genes in activated stroma compared to normal stroma. D) Heat Map of genes correlated with CEACAM6 correlation score of ±>0.5 and P value <0.05. across classical activated and normal stroma.

Online Supplemental Material

Supplementary Figure 5

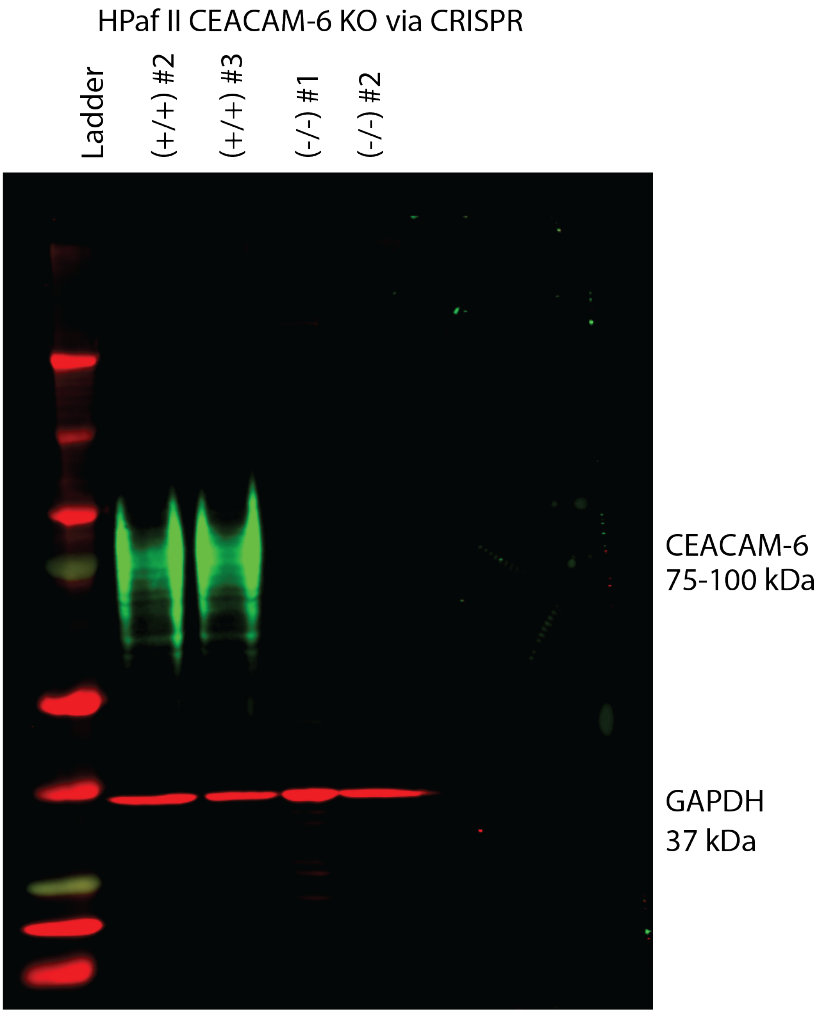

Supplementary Figure 5: Western Blot for confirming CEACAM-6 KO in HPAF-II cells via CRISPR Method

Online Supplemental Material

Supplementary Figure 6 A

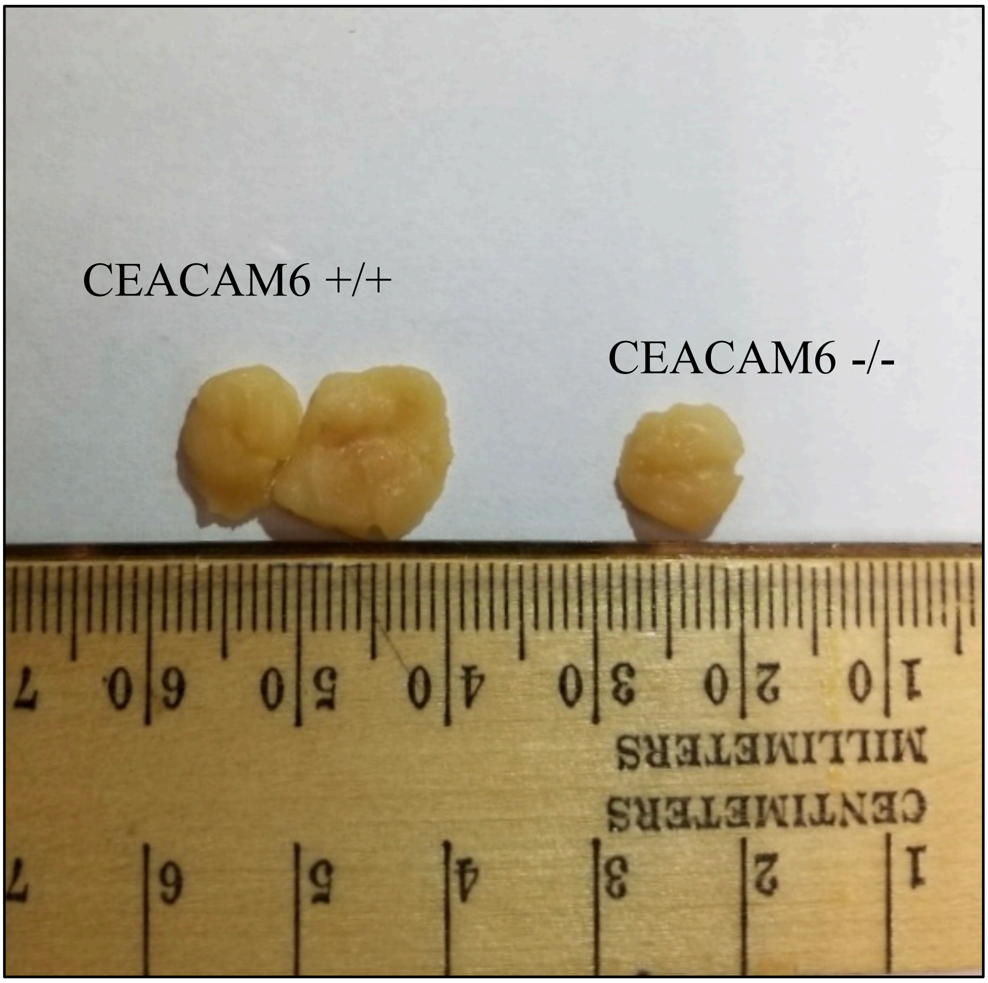

Supplementary Figure 6 A. Image showing size of HPAF-II subcutaneous tumors harvested from CEACAM6 -/- vs. CEACAM6 +/+ mice at the end of the study.

Online Supplemental Material

Supplementary Figure 6 B

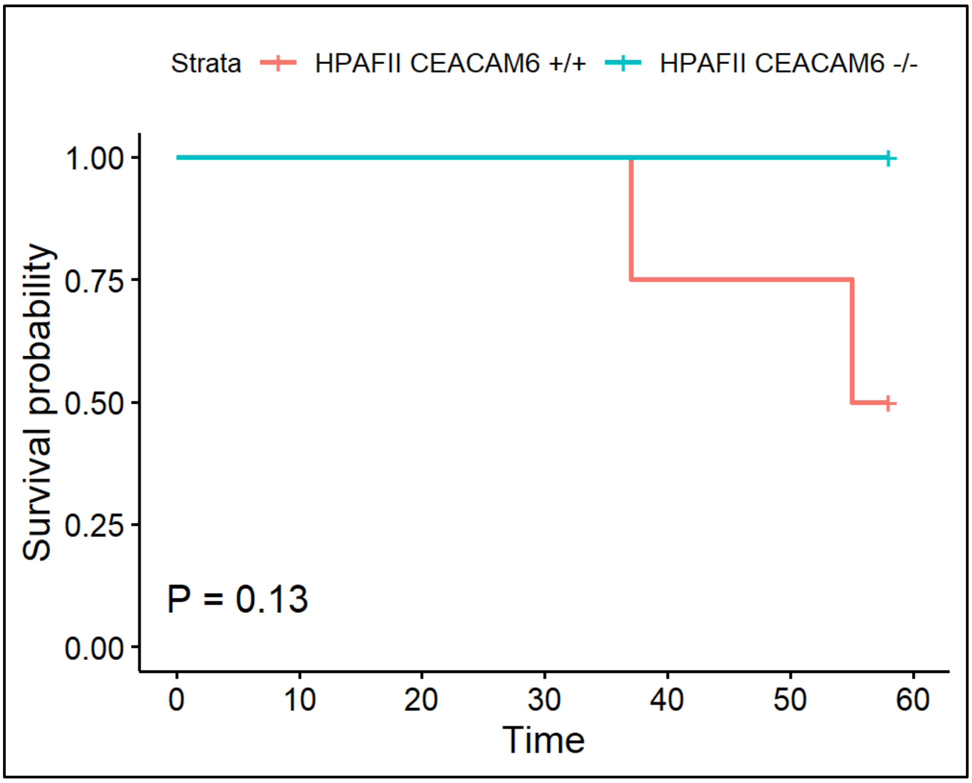

Supplementary Figure 6 C

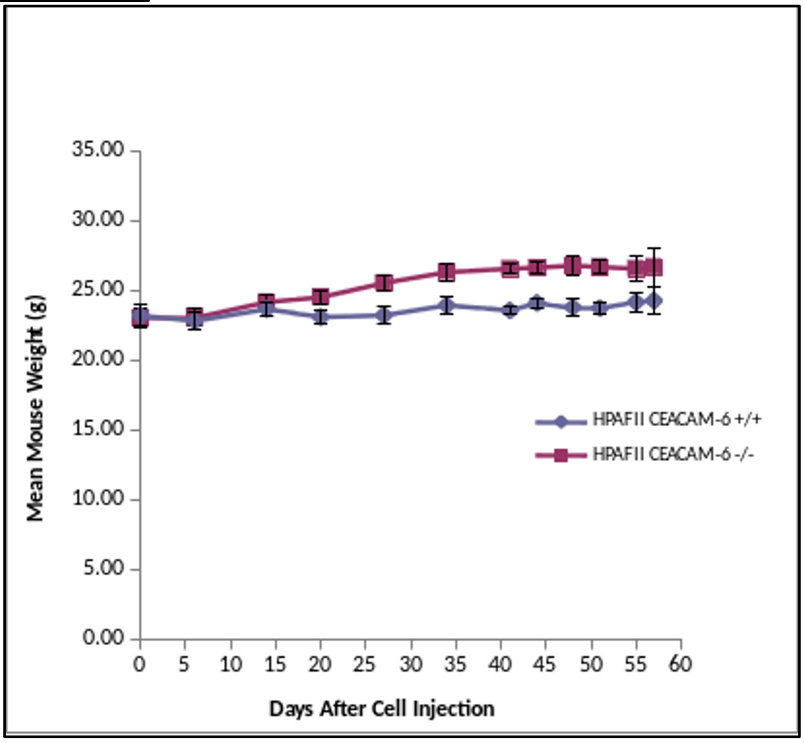

Supplementary Figure 6B. Kaplan-Meier survival curves for mice bearing HPAF-II subcutaneous tumors of CEACAM6 -/- vs. CEACAM6 +/+ shows a difference in overall survival. With n=4 in each group, statistical significance cannot be achieved. **6C**. Body weight changes of mice bearing HPAF-II tumors CEACAM6 -/- vs. CEACAM6 +/+ showed no significant reduction.

Online Supplemental Material

Supplementary Table 1

TCGA RNA-Seq data was investigated for CEACAM6 expression and its association with mutant KRAS and low cytolytic levels in PDA.

		**CEACAM6 Higher 50%**	**CEACAM6 Lower 50%**	**P value**	**CEACAM6 Higher 25%**	**CEACAM6 Lower 75%**	**P value**
**KRAS mutation**	(Mutant)<br>Yes<br>No	76 (89.4%)<br>9 (10.6%)	54 (78.3%)<br>15 (21.7%)	0.09	42 (95.5%)<br>2 (4.5%)	88 (80%)<br>22 (20%)	0.03
**Cytolytic Activity**	(Levels)<br>Higher 50%<br>Lower 50%	4 (15.4%)<br>22 (84.6%)	10 (50%)<br>10 (50%)	0.03	1 (8.3%)<br>11(91.7%)	13 (38.2%)<br>21 (61.8%)	0.07

Supplementary Table 2 - Proteins identified to be altered in CEACAM6 CRISP/Cas9 Knockout HPAF-II cells.

Symbol	logFC	P.Value	Gene ID	Description	Function
THBS3	3.38	0.00395937	7059	thrombospondin 3	*ExtraCellular Matrix, Cell adhesion and Transmembrane proteins*
SPRR3	2.89	0.00178167	6707	small proline rich protein 3	*ExtraCellular Matrix, Cell adhesion and Transmembrane proteins*
MMP1	2.29	0.00098956	4312	matrix metallopeptidase 1	*ExtraCellular Matrix, Cell adhesion and Transmembrane proteins*
PALLD	2.21	0.00964516	23022	palladin, cytoskeletal associated protein	*ExtraCellular Matrix, Cell adhesion and Transmembrane proteins*
TMEM62	1.43	0.00066463	80021	transmembrane protein 62	*ExtraCellular Matrix, Cell adhesion and Transmembrane proteins*
TMEM164	1.42	0.00781271	84187	transmembrane protein 164	*ExtraCellular Matrix, Cell adhesion and Transmembrane proteins*
CDHR2	1.28	0.00100425	54825	cadherin related family member 2	*ExtraCellular Matrix, Cell adhesion and Transmembrane proteins*
CPNE2	1.05	0.00742963	221184	copine 2	*ExtraCellular Matrix, Cell adhesion and Transmembrane proteins*
TMEM132A	1.00	0.00809262	54972	transmembrane protein 132A	*ExtraCellular Matrix, Cell adhesion and Transmembrane proteins*
LOXHD1	0.95	0.00571658	125336	lipoxygenase homology domains 1	*ExtraCellular Matrix, Cell adhesion and Transmembrane proteins*
EPS8L1	0.84	0.00413874	54869	EPS8 like 1	*ExtraCellular Matrix, Cell adhesion and Transmembrane proteins*
SYTL4	0.76	0.00744625	94121	synaptotagmin like 4	*ExtraCellular Matrix, Cell adhesion and Transmembrane proteins*
C1orf210	0.75	0.00078186	149466	chromosome 1 open reading frame 210	*ExtraCellular Matrix, Cell adhesion and Transmembrane proteins*
ANXA3	0.68	0.00117479	306	annexin A3	*ExtraCellular Matrix, Cell adhesion and Transmembrane proteins*
SNTB2	0.66	0.00390382	6645	syntrophin beta 2	*ExtraCellular Matrix, Cell adhesion and Transmembrane proteins*
ITGB6	0.61	0.00340915	3694	integrin subunit beta 6	*ExtraCellular Matrix, Cell adhesion and Transmembrane proteins*
FKBP15	0.52	0.0089552	23307	FK506 binding protein 15	*ExtraCellular Matrix, Cell adhesion and Transmembrane proteins*
SERPINH1	-0.77	0.00741824	871	serpin family H member 1	*ExtraCellular Matrix, Cell adhesion and Transmembrane proteins*
STX2	-1.02	0.00508384	2054	syntaxin 2	*ExtraCellular Matrix, Cell adhesion and Transmembrane proteins*
TMEM40	-1.55	0.00758292	55287	transmembrane protein 40	*ExtraCellular Matrix, Cell adhesion and Transmembrane proteins*
KRT20-KRT40	-2.30	0.0001278	54474	keratin 20	*ExtraCellular Matrix, Cell adhesion and Transmembrane proteins*
CEACAM6	-6.46	1.26E-09	4680	carcinoembryonic antigen related cell adhesion molecule 6	*ExtraCellular Matrix, Cell adhesion and Transmembrane proteins*
TGM2_TGM4	2.61	0.00932386	7052	transglutaminase 2	*Metabolic*
PLA2G4A	2.22	0.00030466	5321	phospholipase A2 group IVA	*Metabolic*
HMOX1	1.73	0.00473689	3162	heme oxygenase 1	*Metabolic*
PTGS2-PTGS1	1.70	0.00317117	5743	prostaglandin-endoperoxide synthase 2	*Metabolic*
AASS	1.46	0.00909247	10157	aminoadipate-semialdehyde synthase	*Metabolic*
TCIRG1	1.44	0.00424704	10312	T cell immune regulator 1, ATPase H+ transporting V0 subunit a3	*Metabolic*
ATP6V0A1	1.30	0.00558834	535	ATPase H+ transporting V0 subunit a1	*Metabolic*
GGPS1	1.14	0.00213313	9453	geranylgeranyl diphosphate synthase 1	*Metabolic*
ABCD1_ABCD2	1.14	0.00232338	215	ATP binding cassette subfamily D member 1	*Metabolic*
SEPHS2	1.11	0.00765713	22928	selenophosphate synthetase 2	*Metabolic*
AMPD3	0.85	0.00915718	272	adenosine monophosphate deaminase 3	*Metabolic*
ASAH1	0.79	0.00070705	427	N-acylsphingosine amidohydrolase 1	*Metabolic*
DGKA-DGKB	0.74	0.00589715	1606	diacylglycerol kinase alpha	*Metabolic*
ATP6V0D1-ATP6V0D2	0.66	0.00601523	9114	ATPase H+ transporting V0 subunit d1	*Metabolic*
TFB2M	0.59	0.00090854	64216	transcription factor B2, mitochondrial	*Metabolic*
POLG	0.55	0.0024374	5428	DNA polymerase gamma, catalytic subunit	*Metabolic*
IARS2	0.55	0.00608626	55699	isoleucyl-tRNA synthetase 2, mitochondrial	*Metabolic*
TALDO1	0.50	0.00197797	6888	transaldolase 1	*Metabolic*
GLS	0.48	0.00940095	2744	glutaminase	*Metabolic*
GFM2	0.47	0.00726989	84340	G elongation factor mitochondrial 2	*Metabolic*

ACBD3	0.45	0.00210669	64746	acyl-CoA binding domain containing 3	*Metabolic*
GSTZ1	-0.50	0.00630706	2954	glutathione S-transferase zeta 1	*Metabolic*
RXRA-RXRG-RXRB	-0.52	0.0085552	6256	retinoid X receptor alpha	*Metabolic*
USP11	-0.58	0.00241228	8237	ubiquitin specific peptidase 11	*Metabolic*
ARL2	-0.62	0.00988356	402	ADP ribosylation factor like GTPase 2	*Metabolic*
SMC6	-0.63	0.0011285	79677	structural maintenance of chromosomes 6	*Metabolic*
MRPS11	-0.68	0.00734965	64963	mitochondrial ribosomal protein S11	*Metabolic*
MRPL23	-0.69	0.00816799	6150	mitochondrial ribosomal protein L23	*Metabolic*
MRPL43	-0.79	0.00721412	84545	mitochondrial ribosomal protein L43	*Metabolic*
PLEKHA4	-0.84	0.0070769	57664	pleckstrin homology domain containing A4	*Metabolic*
APOBEC3B-APOBEC3.	-1.06	0.00751339	200315	apolipoprotein B mRNA editing enzyme catalytic subunit 3A	*Metabolic*
NSMCE2	-1.11	0.00696629	286053	NSE2 (MMS21) homolog, SMC5-SMC6 complex SUMO ligase	*Metabolic*
AGK	-1.36	0.00110123	55750	acylglycerol kinase	*Metabolic*
PHGDH	-1.92	0.00047669	26227	phosphoglycerate dehydrogenase	*Metabolic*
CES1-CES1P1	-2.61	2.99E-05	1066	carboxylesterase 1	*Metabolic*
MAN1A1	-4.14	2.85E-06	4121	mannosidase alpha class 1A member 1	*Metabolic*
IL2RG	3.23	3.73E-05	3561	interleukin 2 receptor subunit gamma	*Immune Related*
ATP6V0A1	1.30	0.00558834	535	ATPase H+ transporting V0 subunit a1	*Immune Related*
IRF6	1.15	0.00935499	3664	interferon regulatory factor 6	*Immune Related*
OSMR	1.08	0.0097181	9180	oncostatin M receptor	*Immune Related*
GBP2	1.05	0.00441838	2634	guanylate binding protein 2	*Immune Related*
NIT1	0.92	0.00728586	4817	nitrilase 1	*Immune Related*
CMIP	0.90	0.00638369	80790	c-Maf inducing protein	*Immune Related*
ARHGAP45-RNF130	0.89	0.00628742	23526	Rho GTPase activating protein 45	*Immune Related*
NFATC2IP	0.72	0.00883435	84901	nuclear factor of activated T cells 2 interacting protein	*Immune Related*
MAL2	0.69	0.00919772	114569	mal, T cell differentiation protein 2 (gene/pseudogene)	*Immune Related*
DAB2IP	0.53	0.00736605	153090	DAB2 interacting protein	*Immune Related*
SERPINB9	0.44	0.00802986	5272	serpin family B member 9	*Immune Related*
TRAF3	-1.23	0.0097234	7187	TNF receptor associated factor 3	*Immune Related*
SIVA1	-1.80	0.00493064	10572	SIVA1 apoptosis inducing factor	*Immune Related*
HMOX1	1.73	0.00473689	3162	heme oxygenase 1	*Transport*
TCIRG1	1.44	0.00424704	10312	T cell immune regulator 1, ATPase H+ transporting V0 subunit a3	*Transport*
ATP6V0A1	1.30	0.00558834	535	ATPase H+ transporting V0 subunit a1	*Transport*
ABCD1_ABCD2	1.14	0.00232338	215	ATP binding cassette subfamily D member 1	*Transport*
ATP13A2	1.13	0.00193599	23400	ATPase cation transporting 13A2	*Transport*
SLC9A3R2	1.13	0.00609537	9351	SLC9A3 regulator 2	*Transport*
ABCC10	0.83	0.00915559	89845	ATP binding cassette subfamily C member 10	*Transport*
MCOLN1	0.72	0.00201328	57192	mucolipin 1	*Transport*
ATP6V0D1-ATP6V0D2	0.66	0.00601523	9114	ATPase H+ transporting V0 subunit d1	*Transport*
USO1	0.56	0.00863236	8615	USO1 vesicle transport factor	*Transport*
SERINC1	0.53	0.00119275	57515	serine incorporator 1	*Transport*
TOM1L2	0.45	0.00894057	146691	target of myb1 like 2 membrane trafficking protein	*Transport*
RXRA-RXRG-RXRB	-0.52	0.0085552	6256	retinoid X receptor alpha	*Transport*
SYTL1	-0.63	0.00987322	84958	synaptotagmin like 1	*Transport*
CLU	-0.69	0.00284476	1191	clusterin (secreted chaperone)	*Transport*

KDELC2	-0.84	0.0028488	143888	KDEL motif containing 2	*Transport*
STXBP6	-2.15	0.00255906	29091	syntaxin binding protein 6	*Transport*
MAN1A1	-4.14	2.85E-06	4121	mannosidase alpha class 1A member 1	*Transport*
ATG4A	2.00	0.00960591	115201	autophagy related 4A cysteine peptidase	*Autophagy*
AKTIP	1.04	0.00439807	64400	AKT interacting protein	*Autophagy*
WDR44	0.83	0.00279693	54521	WD repeat domain 44	*Autophagy*
SH3GLB1	0.69	0.0076018	51100	SH3 domain containing GRB2 like, endophilin B1	*Autophagy*
RANBP10	0.85	0.00164511	57610	RAN binding protein 10	Transcription, chromatin modifying and Dna repair
LIG4	0.70	0.00084696	3981	DNA ligase 4	Transcription, chromatin modifying and Dna repair
MLLT3	0.63	0.00478039	4300	MLLT3, super elongation complex subunit	Transcription, chromatin modifying and Dna repair
ZRANB2	0.58	0.00071819	9406	zinc finger RANBP2-type containing 2	Transcription, chromatin modifying and Dna repair
THUMPD1	0.53	0.00010498	55623	THUMP domain containing 1	Transcription, chromatin modifying and Dna repair
CARS	0.52	0.0070629	833	cysteinyl-tRNA synthetase	Transcription, chromatin modifying and Dna repair
RSRC1	0.49	0.0068122	51319	arginine and serine rich coiled-coil 1	Transcription, chromatin modifying and Dna repair
PUS7L	0.45	0.00821591	83448	pseudouridylate synthase 7 like	Transcription, chromatin modifying and Dna repair
RNASET2	-0.39	0.00661411	8635	ribonuclease T2	Transcription, chromatin modifying and Dna repair
INO80C	-0.44	0.00459046	125476	INO80 complex subunit C	Transcription, chromatin modifying and Dna repair
CIRBP	-0.48	0.00450374	1153	cold inducible RNA binding protein	Transcription, chromatin modifying and Dna repair
SIX4	-0.49	0.00723609	51804	SIX homeobox 4	Transcription, chromatin modifying and Dna repair
MTA1	-0.51	0.00565937	9112	metastasis associated 1	Transcription, chromatin modifying and Dna repair
SETX	-0.52	0.00429936	23064	senataxin	Transcription, chromatin modifying and Dna repair
CSTF2T	-0.53	0.00718038	23283	cleavage stimulation factor subunit 2 tau variant	Transcription, chromatin modifying and Dna repair
MSL1	-0.53	0.00765448	339287	male specific lethal 1 homolog	Transcription, chromatin modifying and Dna repair
CWC15	-0.63	0.00596785	51503	CWC15 spliceosome associated protein homolog	Transcription, chromatin modifying and Dna repair
PRPF40B	-0.63	0.00951814	25766	pre-mRNA processing factor 40 homolog B	Transcription, chromatin modifying and Dna repair
CHD7	-0.74	0.00592792	55636	chromodomain helicase DNA binding protein 7	Transcription, chromatin modifying and Dna repair
RPS13	-0.75	0.00876639	6207	ribosomal protein S13	Transcription, chromatin modifying and Dna repair
NUF2	-0.77	0.00981254	83540	NUF2, NDC80 kinetochore complex component	Transcription, chromatin modifying and Dna repair
DDB2	-0.80	0.0024216	1643	damage specific DNA binding protein 2	Transcription, chromatin modifying and Dna repair
MBTD1	-0.84	0.0068143	54799	mbt domain containing 1	Transcription, chromatin modifying and Dna repair
KANSL3	-0.84	0.0021869	55683	KAT8 regulatory NSL complex subunit 3	Transcription, chromatin modifying and Dna repair
ELMSAN1	-0.90	0.00796766	91748	ELM2 and Myb/SANT domain containing 1	Transcription, chromatin modifying and Dna repair
BRCA1	-0.93	0.00413145	672	BRCA1, DNA repair associated	Transcription, chromatin modifying and Dna repair
RBM38-RBM24	-1.00	0.0027955	55544	RNA binding motif protein 38	Transcription, chromatin modifying and Dna repair
MYNN	-1.21	0.00153659	55892	myoneurin	Transcription, chromatin modifying and Dna repair
CDC40	-1.22	0.0085515	51362		Transcription, chromatin modifying and Dna repair
ICE1	-1.26	0.00858076	23379	interactor of little elongation complex ELL subunit 1	Transcription, chromatin modifying and Dna repair
HASPIN	-1.34	0.00580125	83903	histone H3 associated protein kinase	Transcription, chromatin modifying and Dna repair
ATXN7	-1.91	0.0089362	6314	ataxin 7	Transcription, chromatin modifying and Dna repair
PTK7	0.87	0.00755274	5754	protein tyrosine kinase 7 (inactive)	Signal Transduction
MAP4K4	0.71	0.00479321	9448	mitogen-activated protein kinase kinase kinase kinase 4	Signal Transduction
TAOK2	0.59	0.00182925	9344	TAO kinase 2	Signal Transduction
THAP12	-0.72	2.59E-07	5612	THAP domain containing 12	Signal Transduction
SKIL	-0.95	0.00695193	6498	SKI like proto-oncogene	Signal Transduction
KCTD14	-2.64	0.00784142	65987	potassium channel tetramerization domain containing 14	Signal Transduction

RXRA-RXRG-RXRB	-0.52	0.0085552	6256	retinoid X receptor alpha	Signal Transduction
CPPED1	1.38	0.00564029	55313	calcineurin like phosphoesterase domain containing 1	Miscellaneous proteins
GDAP2	1.17	0.00105523	54834	ganglioside induced differentiation associated protein 2	Miscellaneous proteins
F3	1.08	0.00076506	2152	coagulation factor III, tissue factor	Miscellaneous proteins
WBP2	0.99	0.0086228	23558	WW domain binding protein 2	Miscellaneous proteins
MFSD1	0.91	0.00842618	64747	major facilitator superfamily domain containing 1	Miscellaneous proteins
FCHSD1	0.81	0.00083553	89848	FCH and double SH3 domains 1	Miscellaneous proteins
STBD1	0.80	0.00254307	8987	starch binding domain 1	Miscellaneous proteins
QSOX1	0.75	0.0085823	5768	quiescin sulfhydryl oxidase 1	Miscellaneous proteins
CTBS	0.63	0.00469955	1486	chitobiase	Miscellaneous proteins
RNPEP	0.61	0.00934013	6051	arginyl aminopeptidase	Miscellaneous proteins
PLBD2	0.55	0.0001094	196463	phospholipase B domain containing 2	Miscellaneous proteins
HSPA1A-HSPA7	0.36	0.009654	3303	heat shock protein family A (Hsp70) member 1A	Miscellaneous proteins
GRAMD1B	-0.53	0.00566959	57476	GRAM domain containing 1B	Miscellaneous proteins
HSPA2	-0.59	0.00334339	3306	heat shock protein family A (Hsp70) member 2	Miscellaneous proteins
